# Supplementary material for: A study of the impact of data sharing on article citations using journal policies as a natural experiment
Source: PLoS One. 2019 Dec 18;14(12):e0225883. doi: 10.1371/journal.pone.0225883 (PMC6919593; doi:10.1371/journal.pone.0225883)
Supplement: S1 File — (DOCX) [file pone.0225883.s002.docx]

**Supplemental Online Material for**

***A study of the impact of data sharing on article citations using journal policies as a natural experiment***

**Authors:**

**Garret Christensen** <garret@berkeley.edu> (510) 847-6081, ORCID: 0000-0003-2558-1609

**Allan Dafoe** <allan.dafoe@governance.ai> (203) 350-3750, ORCID: 0000-0003-0377-205X

**Edward Miguel** <emiguel@berkeley.edu> (510) 642-4361, ORCID: 0000-0001-6927-709X

**Don A. Moore** <dm@berkeley.edu> (510) 642-1059, ORCID: 0000-0002-6537-9598

**Andrew K. Rose** <andrewkrose@berkeley.edu> (510) 642-6609, ORCID: 0000-0003-1100-1212

Contents

[Appendix A 2](#_Toc18175695)

[Backstory 2](#_Toc18175696)

[Moore, Rose: Seventeen journals that switched policy 2](#_Toc18175697)

[Identification of Treatment Journals 2](#_Toc18175698)

[Identification of Control Journals 5](#_Toc18175699)

[Model Specification and Estimation 9](#_Toc18175700)

[Benchmark Results 10](#_Toc18175701)

[Sensitivity Analysis 14](#_Toc18175702)

[Heterogeneity Across Journals 19](#_Toc18175703)

[Assessing Data Availability Across our 17 Focal Journals 21](#_Toc18175704)

[Appendix B 25](#_Toc18175705)

[Christensen, Dafoe, Miguel Analysis 25](#_Toc18175706)

[Methods 25](#_Toc18175707)

[Data 25](#_Toc18175708)

[Details of Data Collection in CDM Analysis 26](#_Toc18175709)

[Empirical Methodology 27](#_Toc18175710)

[Results 29](#_Toc18175711)

[Summary Stats 29](#_Toc18175712)

[Naive OLS Results 33](#_Toc18175713)

[Two Stage Least Squares 35](#_Toc18175714)

[Exclusion Restriction 40](#_Toc18175715)

[Different Measures of Availability and Citations 44](#_Toc18175716)

[Results for Data Availability Only 46](#_Toc18175717)

[Results for All Articles and for Political Science Only 52](#_Toc18175718)

[References 58](#_Toc18175719)

# Appendix A

## Backstory

Christensen, Dafoe, and Miguel (CDM) wrote a pre-analysis plan for the political science portion of our project in January 2015. They did so before having gathered the data, so as to bind their hands in the final analysis and reduce their ability to search for statistical significance. The pre-analysis plan is registered on the Open Science Framework, and is available at <https://osf.io/hv97m/.> (See [1] or [2] for a more thorough justification of pre-specifying analyses.) The data ultimately used for the projected was collected from September 2016 to May 2018 by research assistants David Birke, Evey Huang, Baiyue Cao, Terri Cruz, Ravina Pattni, Kevin Khuu, Rachel Kim, Maxim Guzman, Mu Yang Shin, Manana Hakobyan, Don Sun, Neil Tagare, Jacqueline Wood, Isabel Zhou, Kai Ong, and Simon Wu Zhu.

The analysis in the paper largely follows the plan but deviates in small ways. We described our sampling period in the pre-analysis plan, but we did not think to exclude articles that do not use data at that time. Results on the full sample, described in Table B21 to B23, are generally weaker. We also planned to control for time using cubic functions of months since publication. The results presented in the paper instead use year-discipline fixed effects to more flexibly control for time. Models with the cubic function yield nearly identical results.) Note that one of the authors had gathered a subset of the data on data availability previously, without any of the outcome (citation) variables for this study, for use in a 2014 paper [3]. In September 2017 CDM expanded their analysis to include the economics portion of the project.

In February 2017, Moore and Rose (MR) independently developed a plan to study the effects of data posting on citations. In May 2017, they began collecting data with the help of undergraduate research assistants Shreya Agarwal, Amelia Dev, Ekaterina Goncherova, Pranay Kumar, Sydney Mayes, Maya Shen, Cody Strohl, Mitchell Wong, and Winnie Yan. In September 2017, Moore and Rose learned that Christensen, Dafoe, and Miguel were pursuing their project. The two teams met and agreed to keep pursuing our research plans independently. Moore and Rose continued to collect data as they had planned. In January 2018, prior to conducting any data analyses, Moore and Rose posted a pre-analysis plan for how they would test their hypotheses (<https://osf.io/pxdch/>).

## Moore, Rose: Seventeen journals that switched policy

## Identification of Treatment Journals

The first step in the MR analysis identified the scientific journals which began to require the public posting of data. Consistent with our pre-registered research plan we began with the 250 highest-impact scientific journals, as rated by the *Scimago Journal Rank Portal*. The Scimago journal rank indicator measures a scientific journal’s impact much like a traditional impact factor, and attempts to standardize measures across fields to make impact ratings comparable, thus allowing us to examine journals from a wide variety of scientific fields (Guerrero-Bote & Moya-Anegón, 2012). (Scimago is available at http://www.scimagojr.com/journalrank.php; in particular, we use the SJR indicator.)

For each of the 250 journals, we determined whether the journal’s editorial policy included a requirement that authors of published papers share the data supporting the paper’s main findings. Some journals have an editorial policy that requires authors to make a data availability statement (detailing whether or not data is available), but do not make data sharing a requirement; we do not count this as having a required data sharing policy. If data sharing policies were ambiguous (for example, if the policy read something like “we highly encourage authors to share data”), research assistants contacted the editorial staff of the journal for a clarification of the policy. If the editorial staff of a journal replied that data sharing was encouraged but not required, we again do not count that journal as having a required data sharing policy. In total, fifteen journals from the original list of 250 journals qualified as having a required data sharing policy. Our pre-registered analysis plan included one journal (*ACS Nano*) that we subsequently concluded did not actually enforce its data-posting policy. The analyses we present exclude *ACS Nano*, but this decision does not meaningfully change the results.

Additionally, research assistants attempted to identify any other journals with required data sharing policies that were not on the list of the top 250 journals. These web searches included the search phrases “academic journals with required data sharing policies” or “academic journals with required data posting policies.” This identified two additional journals requiring data sharing, leaving us with seventeen “treatment” journals. The treatment journals and the dates when the data sharing requirement was implemented are tabulated in Appendix Table A1.

**Table A1: Treatment Journals with Default Dates for Formal Data Sharing Policy**

| **Treatment Journal** | **Policy Change** | **Control Journal** |
| --- | --- | --- |
| American Economic Review | March 2005 | Journal of Monetary Economics |
| American Journal of Political Science | July 2012 | International Organization |
| American Political Science Review | March 2016 | International Organization |
| BAMS | January 2002 | Criminology |
| Development | February 2011 | EMBO Journal |
| Ecological Applications | January 2014 | Ecology Letters |
| Ecological Monographs | January 2011 | Trends in Ecology and Evolution |
| Econometrica | January 2004 | Journal of Monetary Economics |
| Gastroenterology | July 2007 | PLoS Medicine |
| Journal of Labor Economics | February 2009 | Journal of the European Economic Association |
| Journal of Political Economy | June 2005 | Journal of Financial Economics |
| Methods in Ecology and Evolution | January 2014 | Ecology Letters |
| PLoS Biology | March 2014 | Developmental Cell |
| Political Analysis | January 2012 | International Organization |
| PNAS | October 2005 | Science |
| Review of Economics and Statistics | February 2012 | Journal of Accounting Research |
| Sociological Methods & Research | November 2007 | Journal of Public Admin. Research & Theory |
| **Alternative Dates** |  |  |
| American Journal of Political Science | March 2015 |  |
| BAMS | December 2013 |  |
| Development | February 2016 |  |
| PNAS | April 2015 |  |

Note: BAMS is Bulletin of the American Meteorological Society; PNAS is Proceedings of the National Academy of Sciences of the United States of America

In the case of four treatment journals, there are plausible alternative dates for the shift in data sharing policy; these are usually associated with more stringent enforcement of a pre-existing policy. In such cases, we have both a preferred “default” date for the regime change and a plausible “alternate”; we check explicitly for robustness with respect to these dates in our analysis below. For instance, in the case of AJPS, in July 2012 the editor announced a policy that authors should provide data on Dataverse. However, compliance was not checked or verified, and in March 2015 AJPS tightened replication requirements. BAMS required, in Jan 2002, the “Free and Open Exchange of Environmental Data” while in December 2013 it required “Full and Open Access to Data.” In the case of Development, in February 2011 a policy (posted on the website) required authors to upload micro array data within six months of publication, and to upload gene sequence data at the time of submission. In February 2016, Development formed a partnership with Dryad which required authors to upload micro array and gene sequence data at article submission. PNAS made a data posting requirement in “Information to Authors” in October 2005, but tightened policy in April 2015 by a required data availability statement. For articles published less than five years before the end of the sample (2016), we collected citations for as many years as available.

We are left with a data set of 4,403 empirical papers in our treatment journals using our default policy switch dates (4,491 when our alternate dates are used); each was published close to the date when the relevant treatment journals began to require data sharing. Since each of these papers can contribute up to five individual observations (one for citations in each of up to five years after publication), we end up with a data set of 19,679 observations (using default dates; 16,494 using alternate dates). Since most papers published in our treatment journals are empirical rather than theoretical, the analogous data set for theoretical papers published in the treatment journals has only 3,296 observations (using default dates; 2,812 using alternate dates). By way of contrast, the data set for empirical papers published in our matched control journals has 11,217 observations (using default dates; 9,384 using alternate dates).

In order to determine which of the relevant articles from our journals reported empirical (as opposed to theoretical) research, we chiefly relied on Amazon Mechanical Turk workers (“MTurkers”). A survey instructed the MTurkers how to determine whether an article is empirical, before beginning to classify articles as empirical or not. (The survey is available at https://berkeley.qualtrics.com/jfe/form/SV_0SoVnb1GTvoPgCF?Q_=.) Two MTurkers reviewed each article, and reported whether the article is empirical in nature, as well as its DOI (digital object identifier) and the URL where they found the article. In the (88% of) cases where the answers of the two MTurkers matched, they each received a bonus and we accepted the answer. For the remaining articles, the discrepancy was resolved by research assistants trained to assess the article’s empirical nature.

Throughout, we use a mixture of standard statistical techniques to model citation counts for articles affected and unaffected by data posting requirements. Our data and analysis output are available at <http://faculty.haas.berkeley.edu/arose/RecRes.htm#Misc> and on the OSF at <https://osf.io/pxdch/> .

## Identification of Control Journals

Citations to the *empirical* papers published in our (17) treatment journals are our main interest; we compare citations to papers published just *after* the policy change to citations for papers published just *before*. Citations to *theoretical* papers published in our treatment journals constitute one natural set of “control” observations. We also match our treatment journals to other journals that did *not* switch regime in the sample, as an alternative set of control observations.

Our objective is to identify control journals that do not require data posting, but that are otherwise as similar as possible to the treatment journals in terms of observable characteristics. Accordingly, we selected a set of matched journals (one-to-one matching, with replacement) that have never required data posting from the Scimago “Top 250” list, but are otherwise closely matched journals to our 17 focal journals. The faux intervention for the control journals was set to the same year as the actual policy change for the treated journals, since the objective is to find journals exposed to similar temporal shocks and trends.

Specifically, we chose our set of control journals using conventional propensity score matching. We downloaded the data set used by Scimago to create its rankings (freely available online from http://www.scimagojr.com/journalrank.php). This provides us with the six variables we use to match treatment to control journals: a) the journal’s h-index; b) the total number of citable documents published in the last three years; c) citations/document over the last two years; d) references/document; e) the country where the journal was published; and f) the category of the journal. Since there are only two countries of relevance where our treatment journals were published, we created a binary variable for journals published in the UK, leaving American journals as the default. Since we only have a limited number of treatment journals, we also consolidated journal category into eight areas: Biology; Ecology; Economics; Medicine; Miscellaneous; Molecular Biology; Multidisciplinary; Nano technology; and Sociology and Political Science. Each of the 17 treatment journals was then matched to a control journal within scientific discipline (broadly defined); some control journals were best matches to more than one treatment journal (as a result we had 13 unique journals in our control set).

After creating the data set, we then created a binary variable, with unity for our treatment journals and zero for potential control journals and estimated a cross-sectional probit equation; the results are tabulated below. After estimating the probit model, we then matched each of the treatment journals to a single control journal, using the closest possible journal (by predicted probit score) within journal category. Four of our treatment journals were matched to the same control journal as a previous treatment journal. The control journals are tabulated in Appendix Table A1; the probit regression estimates are tabulated in Appendix Table A2.

**Table A2: Probit Model of Treatment Journal Characteristics**

| h-index | .006  (.003) |
| --- | --- |
| Citable Documents (last three years) | .0000  (.0001) |
| Citations/Document  (last two years) | -.35  (.11) |
| References/Document | -.011  (.009) |
| UK Dummy | .69  (.39) |
| Pseudo R^2^ | .43 |

Regressand is binary variable; 1 for treatment journals, 0 for potential control journals. Estimated with probit; 229 observations; 41 failures completely determined. Estimated tabulated are probit coefficients with standard errors recorded in parentheses. Intercept and (8) journal area controls included but not recorded.

We begin with a simple examination of the data on citations. Many of our papers receive no citations in any given year after publication but a few receive many; we thus examine the natural logarithm of (1+Citations) initially. Descriptive statistics for the six sets of papers are provided in Table A3. For each set of papers, Table 1 provides the minimum, maximum, and mean value of log(1+Citations), as well as the 10^th^, 50^th^ (median), and 90^th^ percentiles of the distribution. These citations include references made any time between the first and fifth years after publication, inclusive. Without exception, statistics for the data *before* the data sharing policy are similar to those corresponding *after* the policy change.

**Table A3: Descriptive Statistics for log(1+Citations)**

| **Journals** | **Papers** | **Before/After**  **Policy Shift** | **Percentile** | | | | | **Mean** |
| --- | --- | --- | --- | --- | --- | --- | --- | --- |
|  |  |  | **Min** | **10** | **50** | **90** | **Max** |  |
| Treatment | Empirical | Before | 0 | 0 | 1.6 | 2.8 | 6.3 | 1.6 |
| Treatment | Empirical | After | 0 | 0 | 1.6 | 3.0 | 6.4 | 1.6 |
| Treatment | Theoretical | Before | 0 | 0 | .7 | 2.2 | 4.4 | 1.0 |
| Treatment | Theoretical | After | 0 | 0 | .7 | 2.2 | 4.4 | 0.9 |
| Control | Empirical | Before | 0 | .7 | 1.8 | 2.8 | 5.5 | 1.7 |
| Control | Empirical | After | 0 | 0 | 1.8 | 3.1 | 5.2 | 1.8 |

Figure A1 provides histograms for each of our six sets of citations. Each of the three columns in Figure A1 represents a combination of journal (either treatment or control) and paper type (either empirical or theoretical); the top row presents the histograms for papers published before the policy change, while those below are the analogues for papers published afterwards. Like the descriptive statistics of Table A3, each histogram portrays the log of (1+Citations) received in any of the first through fifth years after publication. The histograms in the bottom row – representing citations earned *after* data sharing is required – are similar to those above, before the change in policy. The evidence from Table A3 and Figure A1 is informal. Still, it is inconsistent with the notion that citations rise – let alone rise dramatically – with data sharing.

**Figure A1. Histograms of citations of treated empirical, treated theoretical, and control empirical articles, both before and after policy change.**

The latter two sets of data show that citations tend to increase after journal policy shifts towards required data sharing, and this shift verges on statistical significance. This is true for both theoretical papers published in our treatment journals, and empirical articles published in our control journals. That is, where the empirical papers published in our treatment journals do not receive more citations after data sharing is required, both theoretical papers published in the same journals and empirical papers published in our control journals do in fact receive marginally more citations. This implies that the requirement of data sharing might change the nature of publication, raising both the quality of theoretical submissions to treatment journals and the quality of empirical submissions to control journals.

The divergence between average citations before and after the policy shift is small, neither substantively nor statistically significant. This impression is confirmed by the simple *t*-tests for differences in citation means, tabulated in Table A4. The opposite is true for both theoretical papers published in the treatment journals and the empirical papers published in our control journals, which tend to receive more citations after the policy shift. Many of these mean changes are statistically significant; we show below that such evidence fades once we controls are added for other influences.

**Table A4: Simple (t-) Tests for Equality of Citations before and after Regime Change**

| Years since Publication | Empirical Papers, Treatment Journals | Theoretical Papers, Treatment Journals | Empirical Papers, Control Journals |
| --- | --- | --- | --- |
| 1 | -.1 (.2) | .7* (.3) | 1.5*** (.3) |
| 2 | -.4 (.3) | 1.0* (.5) | 1.7*** (.4) |
| 3 | -.5 (.4) | .5 (.5) | 1.6** (.5) |
| 4 | .0 (.5) | -.5 (.5) | 2.0*** (.5) |
| 5 | .1 (.5) | -.4 (.5) | 2.3*** (.6) |

Coefficients are point estimates for (average number of citations for articles published after regime switch) – (average number of citations for articles published before regime switch). Standard error recorded in parentheses; coefficients significantly different from zero at the .05/.01/.005 significance level marked by one/two/three asterisk(s).

## Model Specification and Estimation

We estimate the effect of data posting policies on citations using the following simple model:

Citations_i,j,t+y_ = βPOLICY_j,t_ + γlog(COAUTH)_i,j_ + {φ_j_} + {θ_t_} + u_i,j,t_ for y=1,…,5 (1)

where:

- Citations_i,j,t+y_ is the flow of new citations that article i in journal j published in year t receives in y years after publication,
- β is the coefficient of interest,
- POLICY is a binary variable that is one if journal j required data to be shared in year t and zero otherwise,
- γ is a nuisance coefficient,
- log(COAUTH)_i_ is the natural logarithm of the number of co-authors for article i,
- φ and θ are journal- and (publication-) year-specific fixed effects,
- u is an unexplained residual that accounts for the myriad reasons why citations vary.

Equation (1) estimates the coefficient of interest β, using a “within” panel estimator, since it compared citations to articles in a given journal (received y years after publication) immediately before and after the journal switches data sharing policy, after allowing for fixed effects to account for extraneous factors common to a journal or publication year. It asks the question “What is the effect of required data sharing on the flow of new citations to an article a certain number of years (y) after its publication?” Since we gather citation data for up to five years (if possible) after publication, we estimate (1) in five different cross-sections.

Equation (1) uses citations from each of the five years since publication separately. A different strategy is to pool the data for all years after publication, and estimate the effect of data sharing on citations. We pool across years since publication in two different ways. First, we constrain the effect of the policy shift to be the same – that is, an intercept shift – for all years:

Citations_i,j,t+y_ = βPOLICY_j,t_ + γlog(COAUTH)_i,j_ + {φ_j_} + {θ_t_} + u_i,j,t_ for all y (2)

We also let the effect of the policy change vary over years since publication by estimating:

Citations_i,j,t+y_ = Σ_y_β_y_POLICY_j,t+y_ + γlog(COAUTH)_i,j_ + {φ_j_} + {θ_t_} + u_i,j,t_ for all y (3)

Where (2) forces the effect of the policy shift on citations to be the same for each year after publication, (3) allows the impact to vary by years since publication.

We estimate each of these three models in three ways. First, we estimate (1)-(3) with simple least squares. However the regressand, citations, is far from normally distributed; citations are discrete rather than continuous, plenty of articles receive zero new citations in a given year, and no paper receives a negative number of citations. Accordingly, we also transform the regressand by taking the natural logarithm of one plus citations, and again estimate with least squares. Finally, we estimate the model directly with Poisson regression to account explicitly for the count nature of the regressand. As it turns out, all three estimators deliver qualitatively similar results.

## Benchmark Results

The results in Table A5 tabulate estimates of β from (1), along with its standard error, robust to clustering by journal, treating each of the (five) years since publication separately. There are nine different columns of results. The three at the left-hand side of Table A5 present results from estimating (1) on observations of empirical papers from our set of (17) treatment journals. The three columns in the middle are analogous but consider observations for theoretical papers from the treatment journals, while the three at the right use observations for empirical papers for our control journals. For each of the three different data sets, we present estimates when (1) is estimated: a) with least squares using raw untransformed citations as the regressand; b) with least squares, using as the regressand log(1+citations); and c) with Poisson on raw untransformed citations.

**Table A5: Effect of Policy Switch, treating years after publication separately**

| Years since Publication | Empirical Papers,  Treatment Journals | | | Theoretical Papers,  Treatment Journals | | | Empirical Papers,  Control Journals | | |
| --- | --- | --- | --- | --- | --- | --- | --- | --- | --- |
| Estimator | LS | LS | Poisson | LS | LS | Poisson | LS | LS | Poisson |
| Log(1+Cites) | N | Y | N | N | Y | N | N | Y | N |
| 1 | -.97  (.57) | -.11  (.06) | -.22*  (.09) | .20  (.60) | -.00  (.08) | -.06  (.23) | -2.79  (2.21) | -.19*  (.08) | -.36  (.21) |
| 2 | -.40  (.42) | -.03  (.05) | -.04  (.06) | .93  (.95) | -.04  (.14) | .12  (.18) | -1.28  (1.61) | -.02  (.07) | -.14  (.14) |
| 3 | -.17  (.57) | -.05  (.05) | -.01  (.07) | .27  (.83) | -.01  (.10) | -.04  (.15) | -1.82  (2.14) | -.04  (.07) | -.18  (.16) |
| 4 | .37  (.53) | .01  (.07) | .04  (.06) | -.19  (1.54) | -.03  (.19) | -.06  (.32) | -2.41  (3.02) | -.02  (.12) | -.23  (.19) |
| 5 | .18  (.66) | .00  (.06) | .02  (.07) | -.79  (1.18) | -.10  (.13) | -.17  (.24) | -2.27  (2.40) | -.05  (.09) | -.22  (.14) |

Coefficient on dummy variable (1 for after data posting required, 0 otherwise) estimated with least squares (LS) or Poisson; each cell represents a separate regression. Robust standard error (clustered by journal) recorded in parentheses; coefficients significantly different from zero at the .05 (.01) significance level marked by one (two) asterisk(s). Regressand is annual citations/article unless marked. Controls included but not recorded: intercept, fixed effects for journal and publication year, and log number of co-authors.

Results from our focal (empirical/treatment) data set are displayed on the left part of Table A5. The most striking result is the predominance of negative estimates of β; empirical papers published after a journal begins to require data sharing tend to receive *fewer* citations for the first three years after publication, compared with empirical papers published in the same journals immediately before the policy shift. Of the fifteen (=three estimators x five time horizon) point estimates, nine are negative. However, the effects are insubstantial and only one of the fifteen estimates is significantly different from zero at even the 5% confidence level. The finding of no statistically significant or substantive effect on citations also characterizes theoretical papers published in treatment journals (tabulated in the middle columns of Table A5) as well as empirical papers published in control journals (in the right-hand columns). Still, the most interesting result is that there are no strong indications of any *positive* effect of data sharing on citations to the empirical papers published in our treatment journals. Our estimates of γ, the effect of the number of co-authors on citations, are consistently positive. We consider this to be a control rather than a coefficient of interest, but are reassured by its stability; this indicates to us that are methodology does not preclude a positive finding, should it be present in the data.

The same message emerges when we pool the data across years since publication; Table A6 presents those estimates. Results of our key coefficient, β, estimated with (2) are tabulated in the top part of Table A6, while results from (3) appear in the bottom panel. Pooling the data also results in no consistent significant patterns across estimators. This is true of the empirical articles published in our treatment journals (results tabulated at the left), as well as the theoretical articles published in the treatment journals and the empirical articles published in our control journals (tabulated in the middle and right columns of Table A6 respectively). Still, the presence of absence remains the most striking feature of the key results at the left; there is no evidence that requiring data sharing boosts citations. Indeed, the data points, if anything, weakly in the opposite direction.

**Table A6: Effect of Policy Switch, treating years after publication simultaneously**

| Years since Publication | Empirical Papers,  Treatment Journals | | | Theoretical Papers,  Treatment Journals | | | Empirical Papers,  Control Journals | | |
| --- | --- | --- | --- | --- | --- | --- | --- | --- | --- |
| Estimator | LS | LS | Poisson | LS | LS | Poisson | LS | LS | Poisson |
| Log(1+Cites) | N | Y | N | N | Y | N | N | Y | N |
| 1-5 | -.20  (.42) | -.03  (.05) | -.02  (.06) | .11  (.88) | -.04  (.11) | -.04  (.20) | -1.98  (2.15) | -.06  (.08) | -.21  (.16) |
|  |  |  |  |  |  |  |  |  |  |
| 1 | -.08  (.59) | -.00  (.07) | -.00  (.10) | .09  (.98) | -.01  (.14) | .03  (.22) | -2.14  (2.51) | -.05  (.08) | -.20  (.20) |
| 2 | -.17  (.23) | -.03  (.03) | -.02  (.03) | .18  (.48) | -.02  (.05) | .00  (.10) | -.93  (1.00) | -.03  (.04) | -.10  (.07) |
| 3 | -.14  (.16) | -.02  (.02) | -.02  (.02) | .10  (.27) | -.00  (.03) | -.01  (.06) | -.64  (.68) | -.02  (.02) | -.07  (.05) |
| 4 | .04  (.14) | .01  (.01) | .01  (.02) | .01  (.22) | -.01  (.03) | -.01  (.05) | -.45  (.54) | -.01  (.02) | -.05  (.04) |
| 5 | -.06  (.10) | -.01  (.01) | -.01  (.01) | -.06  (.17) | -.02  (.02) | -.03  (.04) | -.43  (.41) | -.02  (.02) | -.05  (.03) |

Coefficient on dummy variable (1 for after data posting required, 0 otherwise) estimated with least squares (LS) or Poisson; each column represents a separate regression. Robust standard error (clustered by journal) recorded in parentheses; coefficients significantly different from zero at the .05/.01.005 significance level marked by one/two/three asterisk(s). Regressand is annual citations/article. Controls included but not recorded: intercepts for each year elapsed since publication, fixed effects for journal and publication year, and log number of co-authors.

Thus far, our analysis has considered all citation levels similarly; our statistical analysis quantifies the effect of a formal data sharing policy on the level (or log) of citations. In Tables A7 and A8, we transform the regressand to consider whether data posting changes the odds of an empirical paper being either a “dog” with a small number of citations or a “hit” with a high number, if it is published in a treatment journal with a data sharing policy. In the extreme left-hand column, we consider a paper to be a dog if it receives no new citations at all. We create an appropriate binary regressand (one if the paper receives no new citations in a given year, zero if it receives any) and re-estimate (1)-(3), using probit to account for the binary nature of the dependent variable. Most of the estimates of β are positive, indicating that the probability of publishing a dog is higher after a data sharing policy is imposed, though not to a statistically significant effect degree. To check on the robustness of our definition of a dog, we use three different thresholds, defining a paper as a dog if it receives either: a) no new citations in a year; b) either no or one new citation; or c) less than five new citations. Similarly, we define hits as paper that receive: a) a flow of at least ten new citations in a year; b) at least twenty-five new citations; or c) at least fifty new citations. The results in Tables A7 and A8 indicate that the probability of a dog/hit being published after a switch in data policy tend to rise/fall, though rarely to any significant degree. Again, there is no evidence of increased citations following a data sharing policy.

**Table A7: Effect of Policy Switch, treating years after publication separately, controlling for number of co-authors, empirical papers in treatment journals, dogs and hits**

| Years since Publication | 0  Cites | 0-1  Cites | 0-4  Cites | >=10  Cites | >=25  Cites | >=50  Cites |
| --- | --- | --- | --- | --- | --- | --- |
| 1 | .09  (.06) | .05  (.07) | .14  (.09) | -.34*  (.16) | -.50***  (.15) | .12  (.16) |
| 2 | .00  (.08) | .00  (.08) | .02  (.06) | -.02  (.07) | -.19  (.17) | -.46*  (.21) |
| 3 | .21***  (.06) | .14***  (.05) | -.05  (.10) | -.01  (.07) | -.19  (.20) | -.18  (.16) |
| 4 | -.06  (.17) | -.05  (.12) | -.06  (.06) | -.11  (.07) | .01  (.13) | -.23*  (.09) |
| 5 | -.09  (.18) | -.05  (.09) | -.06  (.07) | .01  (.07) | -.22***  (.08) | -.20  (.12) |

Coefficient on dummy variable (1 for after data posting required, 0 otherwise) estimated with probit; each cell represents a separate regression. Robust standard error (clustered by journal) recorded in parentheses; coefficients significantly different from zero at the .05/.01.005 significance level marked by one/two/three asterisk(s). Regressand is 1 for specified citations, 0 otherwise. Controls included but not recorded: intercept, log number of co-authors, fixed effects for journal and publication year. Default journal regime shift dates.

**Table A8: Effect of Policy Switch, treating years after publication simultaneously, controlling for number of co-authors, empirical papers in treatment journals, dogs and hits**

| Years since Publication | 0  Cites | 0-1  Cites | 0-4  Cites | >=10  Cites | >=25  Cites | >=50  Cites |
| --- | --- | --- | --- | --- | --- | --- |
| 1-5 | .04  (.07) | .02  (.04) | -.02  (.06) | -.07  (.06) | -.17  (.11) | -.22  (.12) |
|  | | | | | | |
| 1 | .01  (.07) | -.02  (.05) | -.08  (.10) | -.06  (.13) | -.34  (.18) | -.21  (.32) |
| 2 | .04  (.04) | .03  (.03) | .02  (.03) | -.01  (.04) | -.03  (.09) | -.22*  (.09) |
| 3 | .05  (.03) | .02  (.02) | .00  (.02) | -.04  (.02) | -.07  (.05) | -.04  (.04) |
| 4 | -.02  (.02) | -.02  (.02) | -.02  (.02) | -.01  (.02) | -.02  (.03) | -.04  (.02) |
| 5 | .01  (.02) | .01  (.01) | .00  (.01) | -.02  (.01) | -.05***  (.02) | -.05  (.03) |

Coefficient on dummy variable (1 for after data posting required, 0 otherwise) estimated with probit; each column represents a separate regression. Robust standard error (clustered by journal) recorded in parentheses; coefficients significantly different from zero at the .05/.01.005 significance level marked by one/two/three asterisk(s). Regressand is 1 for specified citations, 0 otherwise; data set includes empirical papers from treatment journals. Controls included but not recorded: log number of co-authors, intercepts for each year elapsed since publication, fixed effects for journal and publication year. Default journal regime shift dates.

## Sensitivity Analysis

We now show that our results do not depend on a number of the key methodological choices we made in the results presented above.

Tables A9 and A10 use our default dates for the four treatment journals where we are uncertain about the exact date when data sharing was required. Tables A9 and A10 are analogues to Tables A5 and A6 but use our alternate dates. In the large, our results are unchanged; the alternate dates provide no evidence that articles published in journals that require data sharing receive more citations when the policy is in place. Indeed, the alternate dates provide, if anything, marginal evidence that required data posting seems to *reduce* citations slightly in the first year after publication.

**Table A9: Effect of Policy Switch, treating years after publication separately**

**Alternate Policy Switch Dates**

| Years since Publication | Empirical Papers,  Treatment Journals | | | Theoretical Papers,  Treatment Journals | | | Empirical Papers,  Control Journals | | |
| --- | --- | --- | --- | --- | --- | --- | --- | --- | --- |
| Estimator | LS | LS | Poisson | LS | LS | Poisson | LS | LS | Poisson |
| Log(1+Cites) | N | Y | N | N | Y | N | N | Y | N |
| 1 | -1.04*  (.39) | -.11*  (.05) | -.15*  (.07) | -.59  (1.15) | .00  (.09) | -.21  (.29) | -1.37  (.63) | -.17**  (.05) | -.16**  (.03) |
| 2 | -.32  (.41) | -.04  (.04) | -.03  (.05) | -.49  (1.26) | -.00  (.14) | -.12  (.23) | -.55  (1.21) | .05  (.07) | -.06  (.09) |
| 3 | -.26  (1.26) | .02  (.08) | .00  (.15) | -.38  (.94) | .01  (.13) | -.09  (.19) | .56  (.38) | .06  (.05) | .08*  (.03) |
| 4 | -.95  (1.54) | .03  (.09) | -.08  (.16) | -1.92  (1.02) | -.19  (.18) | -.48*  (21) | 1.35  (.61) | .15  (.11) | .13  (.06) |
| 5 | -1.64  (1.88) | .04  (.12) | -.14  (.19) | -2.26*  (1.00) | -.18  (.12) | -.50**  (.14) | .68  (.60) | .05  (.10) | .03  (.08) |

Coefficient on dummy variable (1 for after data posting required, 0 otherwise) estimated with least squares (LS) or Poisson; each cell represents a separate regression. Robust standard error (clustered by journal) recorded in parentheses; coefficients significantly different from zero at the .05 (.01) significance level marked by one (two) asterisk(s). Regressand is annual citations/article unless marked. Controls included but not recorded: intercept, fixed effects for journal and publication year, and log number of co-authors.

**Table A10: Effect of Policy Switch, treating years after publication simultaneously**

**Alternate Policy Switch Dates**

| Years since Publication | Empirical Papers,  Treatment Journals | | | Theoretical Papers,  Treatment Journals | | | Empirical Papers,  Control Journals | | |
| --- | --- | --- | --- | --- | --- | --- | --- | --- | --- |
| Estimator | LS | LS | Poisson | LS | LS | Poisson | LS | LS | Poisson |
| Log(1+Cites) | N | Y | N | N | Y | N | N | Y | N |
| 1-5 | -.71  (.68) | -.03  (.05) | -.07  (.09) | -.99  (.84) | -.05  (.11) | -.24  (.17) | -.10  (.62) | .01  (.06) | -.03  (.06) |
|  |  |  |  |  |  |  |  |  |  |
| 1 | -.46  (.62) | -.02  (.05) | -.06  (.08) | -1.21  (1.00) | -.07  (.14) | -.21  (.24) | -.06  (.60) | -.03  (.06) | -.11  (.10) |
| 2 | -.33  (.31) | -.04  (.03) | -.04  (.04) | -.45  (.48) | -.02  (.05) | -.10  (.09) | -.19  (.49) | .02  (.04) | .00  (.03) |
| 3 | -.37  (.31) | -.02  (.02) | -.04  (.04) | -.31  (.24) | -.02  (.03) | -.09*  (.04) | .05  (.16) | .02  (.02) | .01  (.01) |
| 4 | -.13  (.24) | .02  (.02) | -.00  (.03) | -.18  (.19) | .00  (.03) | -.05  (.05) | .07  (.16) | .01  (.02) | .01  (.02) |
| 5 | -.23  (.18) | .00  (.01) | -.02  (.02) | -.22  (.15) | -.01  (.02) | -.07  (.04) | -.09  (.09) | -.01  (.02) | -.02  (.01) |

Coefficient on dummy variable (1 for after data posting required, 0 otherwise) estimated with least squares (LS) or Poisson; each column represents a separate regression. Robust standard error (clustered by journal) recorded in parentheses; coefficients significantly different from zero at the .05/.01.005 significance level marked by one/two/three asterisk(s). Regressand is annual citations/article. Controls included but not recorded: intercepts for each year elapsed since publication, fixed effects for journal and publication year, and log number of co-authors.

Table A11 provides extensive further sensitivity analysis. We analyze the robustness of β estimates from (2), using the empirical/treatment observations, since these can be succinctly reported and are indicative of the general thrust of Tables A5 and A6. The top row of Table A11 is the default set of estimates, one each for least squares regression on citations and log(1+citations), and a third for Poisson estimation of citations, all taken from Table A6. The default estimates show a negative but statistically insignificant effect of data sharing on citations for all three estimation techniques.

The first four rows of sensitive analysis in Table A11 change the specification of (2) by successively dropping: a) the control for the log of the number of co-authors (i.e., setting γ=0); b) the journal fixed effects (i.e., setting {φ_j_}=0); c) the publication year fixed effects (i.e., setting {θ_t_}=0); and d) both the journal and publication year fixed effects, which are replaced by interactions between journal and publication year fixed effects. None of this has much impact; the estimates of β remain negative and statistically insignificantly different from zero for all three estimators.

**Table A11: Sensitivity Analysis**

|  | **Least Squares** | **LS on log(1+Citations)** | **Poisson** |
| --- | --- | --- | --- |
| Default | -.20  (.42) | -.03  (.05) | -.02  (.06) |
| Drop log number co-authors | -.17  (.42) | -.03  (.05) | -.02  (.06) |
| Drop journal fixed effects | -.62  (.48) | -.07  (.04) | -.08  (.07) |
| Drop publication year fixed effects | -.08  (.32) | .01  (.03) | -.01  (.05) |
| Substitute journal x year fixed effects | -.40  (.45) | -.06  (.05) | -.05  (.05) |
| Drop first quarter of journals by name | -.17  (.44) | -.04  (.05) | -.02  (.06) |
| Drop last quarter of journals by name | .12  (.73) | .03  (.05) | .03  (.11) |
| Drop first quarter of sample by year | -.15  (.44) | -.03  (.05) | -.01  (.06) |
| Drop last quarter of sample by year | -.16  (.45) | -.03  (.05) | -.02  (.06) |
| Drop Economics journals | .26  (.44) | -.01  (.06) | .03  (.06) |
| Drop Political Science journals | -.40  (.45) | -.05  (.05) | -.05  (.06) |
| Drop Economics and Political Science journals | -.05  (.38) | -.05  (.06) | -.01  (.04) |
| Negative Binomial |  |  | -.03  (.07) |
| Drop 2σ residual outliers | -.23  (.30) | -.02  (.03) |  |
| Drop 2.5σ residual outliers | -.24  (.36) | -.03  (.04) |  |
| Drop 3σ residual outliers | -.35  (.40) | -.03  (.05) |  |
| Drop 0 citation observations |  |  | -.01  (.05) |
| Drop 0/1 citation observations |  |  | -.02  (.05) |
| Drop >14 citation observations |  |  | .01  (.03) |
| Drop >49 citation observations |  |  | -.05  (.06) |

Coefficient on dummy variable (1 for after data posting required, 0 otherwise) estimated with least squares/Poisson; each cell represents a separate regression. Robust standard error (clustered by journal) recorded in parentheses; coefficients significantly different from zero at the .05/.01.005 significance level marked by one/two/three asterisk(s). Regressand is annual citations/article except for middle column; data set includes empirical papers from treatment journals. Controls included but not recorded unless otherwise noted: log number of co-authors, intercepts for each year elapsed since publication, fixed effects for journal and publication year.

The next seven rows of Table A10 estimate equation (2) for smaller observation sets. Successively, the rows drop: a) the first and b) last quarter of the journals by name; c) the first and d) last quarter of the sample by year; e) all Economics journals; f) all Political Science journals; and finally g) all Economics and Political Science journals. A row then replaces Poisson with Negative Binomial estimation. None of these changes to the sample alters the conclusion; the estimates of β remain small and insignificantly different from zero.

The final set of checks removes extreme values from the data set. First, three rows drop observations from the least squares estimates with residuals that lie at least a) two; b) two and a half; and c) three standard deviations from zero. Then, four different sets of observations are dropped from the Poisson estimation, two sets each of small (0 and 0-1) and large (>14 and >49) flows of citations. Throughout essentially all the sensitivity analysis, the point estimates of β remain negative but statistically insignificant.

The estimates in Tables A5-A6 handle three different data sets separately: a) empirical papers in treatment journals (our focus); b) theoretical papers in treatment journals; and c) empirical papers in control journals. In Appendix Tables A12 and A13, we pool the first (empirical/treatment) data set with both of the latter data sets in sequence. This amounts to a “difference-in-difference” estimator of β, since we are comparing, *ceteris paribus*, the effect of required data sharing before and after the policy shift, for papers published in journals that were either affected or unaffected by the new policy regime. Since we have two different sets of control data, we present two sets of estimates. The results tabulated in Tables A5 and A6 lead one to suspect that combining the (usually insignificant and small) results from the empirical/treatment data with the (similarly insignificant and small) results from either control data set, would be expected to deliver pooled estimates akin to the empirical/treatment data. So it turns out; empirical articles affected by the policy shift experience somewhat lower citations than those articles unaffected by a policy shift (either because they do not use data or because they are published in journals that do not require data sharing). Tables A12 and A13 show that this effect is most pronounced immediately after publication but is rarely statistically significant, and is sensitive to the exact statistical technique and data comparison. Critically though, there is no evidence that authors of empirical articles published when data sharing is required are rewarded with higher citations.

**Table A12: Effect of Policy Switch, treating years after publication separately,**

**difference in difference analysis**

| Comparison Set | Theoretical Papers,  Treatment Journals | | | Empirical Papers,  Control Journals | | |
| --- | --- | --- | --- | --- | --- | --- |
| Years since Publication |  |  |  |  |  |  |
| Estimator | LS | LS | Poisson | LS | LS | Poisson |
| Log(1+Cites) | N | Y | N | N | Y | N |
| 1 | -.84  (.53) | -.10  (.05) | -.21*  (.09) | -1.65  (.90) | -.14**  (.05) | -.36  (.20) |
| 2 | -.26  (.39) | -.03  (.05) | -.03  (.06) | -.74  (.66) | -.03  (.04) | -.14  (.14) |
| 3 | -.13  (.51) | -.04  (.05) | -.01  (.07) | -.79  (.88) | -.04  (.04) | -.18  (.16) |
| 4 | .32  (.49) | .00  (.06) | .04  (.06) | -.51  (1.08) | -.00  (.06) | -.23  (.19) |
| 5 | .09  (.61) | -.01  (.05) | .01  (.07) | -.60  (.97) | -.01  (.05) | -.22  (.14) |

Coefficient on dummy variable (1 for after data posting required, 0 otherwise) estimated with least squares/Poisson; each cell represents a separate regression. Robust standard error (clustered by journal) recorded in parentheses; coefficients significantly different from zero at the .05 (.01) significance level marked by one (two) asterisk(s). Regressand is annual citations/article unless marked. Controls included but not recorded: a) intercept, fixed effects for b) journal and c) publication year, d) log number of co-authors, and interactions of a)-d) with comparison set dummy.

**Table A13: Effect of Policy Switch, treating years after publication simultaneously,**

**difference in difference analysis**

| Comparison Set | Theoretical Papers,  Treatment Journals | | | Empirical Papers,  Control Journals | | |
| --- | --- | --- | --- | --- | --- | --- |
| Years since Publication |  |  |  |  |  |  |
| Estimator | LS | LS | Poisson | LS | LS | Poisson |
| Log(1+Cites) | N | Y | N | N | Y | N |
| 1-5 | -.17  (.38) | -.05  (.04) | -.03  (.05) | -.84  (.84) | -.04  (.04) | -.21  (.16) |
|  |  |  |  |  |  |  |
| 1 | -.07  (.52) | -.01  (.06) | .00  (.09) | -.82  (.98) | -.02  (.05) | -.09  (.12) |
| 2 | -.13  (.21) | -.04  (.02) | -.02  (.03) | -.45  (.40) | -.03  (.02) | -.06  (.04) |
| 3 | -.11  (.14) | -.02  (.02) | -.02  (.02) | -.32  (.28) | -.02  (.01) | -.04  (.03) |
| 4 | .03  (.12) | .00  (.01) | .01  (.02) | -.12  (.22) | -.00  (.01) | -.02  (.03) |
| 5 | -.06  (.08) | -.02*  (.01) | -.01  (.01) | -.19  (.16) | -.01  (.01) | -.03  (.02) |

Coefficient on dummy variable (1 for after data posting required, 0 otherwise) estimated with least square/Poisson; each column represents a separate regression. Robust standard error (clustered by journal) recorded in parentheses; coefficients significantly different from zero at the .05/.01.005 significance level marked by one/two/three asterisk(s). Regressand is annual citations/article. Controls included but not recorded: a) intercept, fixed effects for b) journal and c) publication year, d) log number of co-authors, e) years elapsed since publication and interactions of b)-e) with comparison set dummy.

The sensitivity analysis indicates that our results are robust, and do not stem from a minor assumption implicit in our methodology or a small subset of our data. Journals that require data posting do not seem to publish papers that are unusually highly cited.

## Heterogeneity Across Journals

Where the sensitivity analysis of Table A11 combines data from all seventeen treatment journals, Table A14 presents individual results when β is estimated with (2) using data on empirical papers from a single journal. (With an intercept replacing the now-irrelevant journal fixed effects.) There are twenty-one rows, one for each of the thirteen treatment journals with a clear date for the policy change, and two for the four journals with alternate dates. As in Tables A16 and A11, we present three estimates of β, one each for LS on citations and log(1+citations), and a third for Poisson estimation of citations.

There is considerable heterogeneity in the effects across journals. For ten journals, the estimates are either statistically small or inconsistent across estimators. Indeed, β is insignificantly different from zero for all three estimators for seven of these ten journals. Four journals (*American Political Science Review, Journal of Political Economy, Methods in Ecology and Evolution*, and *Sociological Methods & Research*) show consistently *negative* effects of the data sharing policy on citations for all three estimators. *Development* shows consistently positive estimates of β using our default dates, but consistently negative estimates using our alternate dates. Only two journals (*American Economic Review* and *Journal of Labor Economics*) deliver consistently positive estimates of β at conventional significance levels.

**Table A14: Effect of Policy Switch, treating years after publication simultaneously,**

**journal by journal**

| **Journal** (Default Regime Switch Dates) | **LS** | **LS, log(1+Cites)** | **Poisson** |
| --- | --- | --- | --- |
| American Economic Review | 1.08***  (.37) | .19***  (.06) | .26***  (.09) |
| American Journal of Political Science  (default) | -.42  (.47) | -.09  (.09) | -.09  (.10) |
| American Political Science Review | -1.58*  (.75) | -.49*  (.24) | -.76*  (.31) |
| Bulletin of the American Meteorological Society (default) | -.36  (.79) | -.41***  (.13) | -1.24***  (.31) |
| Development (default) | 1.05**  (.40) | .12*  (.06) | .19*  (.08) |
| Ecological Applications | -.50  (.45) | -.09  (.09) | -.11  (.11) |
| Ecological Monographs | -.34  (1.05) | .02  (.11) | -.05  (.15) |
| Econometrica | 1.07  (1.09) | .46***  (.16) | .22  (.20) |
| Gastroenterology | .53  (.87) | .06  (.06) | .05  (.09) |
| Journal of Labor Economics | 2.35***  (.64) | .31*  (.15) | .69***  (.18) |
| Journal of Political Economy | -8.69**  (3.19) | -.42***  (.14) | -.86***  (.22) |
| Methods in Ecology and Evolution | -3.44***  (1.20) | -.40***  (.09) | -.63***  (.17) |
| PLoS Biology | .45  (.80) | -.04  (.09) | .05  (.10) |
| Political Analysis | -1.43  (1.60) | -.10  (.14) | -.27  (.32) |
| Proceedings of the National Academy of Sciences (default) | -.51  (.95) | -.13***  (.04) | -.06  (.10) |
| Review of Economics and Statistics | .60  (.33) | .05  (.05) | .16  (.08) |
| Sociological Methods & Research | -2.32***  (.75) | -.43*  (.21) | -1.12***  (.38) |
| Alternate Regime Switch Dates |  |  |  |
| American Journal of Political Science  (alternate) | -.78  (1.22) | -.16  (.17) | -.16  (.25) |
| Bulletin of the American Meteorological Society (alternate) | 1.81  (3.76) | -.02  (.32) | .17  (.33) |
| Development (alternate) | -1.32***  (.30) | -.30***  (.06) | -.40***  (.09) |
| Proceedings of the National Academy of Sciences (alternate) | -.43  (.82) | -.06  (.05) | -.04  (.08) |

Coefficient on dummy variable (1 for any [1-5] year after data posting required, 0 otherwise) estimated with least square/Poisson; each cell represents a separate regression. Robust standard error recorded in parentheses; coefficients significantly different from zero at the .05/.01/.005 significance level marked by one/two/three asterisk(s). Regressand is annual citations/article unless marked otherwise; data set includes empirical papers from treatment journals. Controls included but not recorded: intercept, log number of co-authors, fixed effects for publication year and years since publication.

**Table A15: Probit Model of Treatment Journal Characteristics**

| h-index | .006  (.003) |
| --- | --- |
| Citable Documents (last three years) | .0000  (.0001) |
| Citations/Document  (last two years) | -.35  (.11) |
| References/Document | -.011  (.009) |
| UK Dummy | .69  (.39) |
| Pseudo R^2^ | .43 |

Regressand is binary variable; 1 for treatment journals, 0 for potential control journals. Estimated with probit; 229 observations; 41 failures completely determined. Estimated tabulated are probit coefficients with standard errors recorded in parentheses. Intercept and (8) journal area controls included but not recorded.

**Table A16: Effect of Policy Switch, treating years after publication separately**

**Alternate Policy Switch Dates**

| Years since Publication | Empirical Papers,  Treatment Journals | | | Theoretical Papers,  Treatment Journals | | | Empirical Papers,  Control Journals | | |
| --- | --- | --- | --- | --- | --- | --- | --- | --- | --- |
| Estimator | LS | LS | Poisson | LS | LS | Poisson | LS | LS | Poisson |
| Log(1+Cites) | N | Y | N | N | Y | N | N | Y | N |
| 1 | -1.04*  (.39) | -.11*  (.05) | -.15*  (.07) | -.59  (1.15) | .00  (.09) | -.21  (.29) | -1.37  (.63) | -.17**  (.05) | -.16**  (.03) |
| 2 | -.32  (.41) | -.04  (.04) | -.03  (.05) | -.49  (1.26) | -.00  (.14) | -.12  (.23) | -.55  (1.21) | .05  (.07) | -.06  (.09) |
| 3 | -.26  (1.26) | .02  (.08) | .00  (.15) | -.38  (.94) | .01  (.13) | -.09  (.19) | .56  (.38) | .06  (.05) | .08*  (.03) |
| 4 | -.95  (1.54) | .03  (.09) | -.08  (.16) | -1.92  (1.02) | -.19  (.18) | -.48*  (21) | 1.35  (.61) | .15  (.11) | .13  (.06) |
| 5 | -1.64  (1.88) | .04  (.12) | -.14  (.19) | -2.26*  (1.00) | -.18  (.12) | -.50**  (.14) | .68  (.60) | .05  (.10) | .03  (.08) |

Coefficient on dummy variable (1 for after data posting required, 0 otherwise) estimated with least squares (LS) or Poisson; each cell represents a separate regression. Robust standard error (clustered by journal) recorded in parentheses; coefficients significantly different from zero at the .05 (.01) significance level marked by one (two) asterisk(s). Regressand is annual citations/article unless marked. Controls included but not recorded: intercept, fixed effects for journal and publication year, and log number of co-authors.

## Assessing Data Availability Across our 17 Focal Journals

Our results made us wonder about enforcement of, and compliance with journals’ data-posting policies. In an attempt to estimate the rate of data posting for each of our seventeen focal journals, we sought to code data availability for a sample of articles from our seventeen focal journals. We planned to have research assistants code as many articles as possible over the course of a summer semester. To generate our list of articles, we randomly sampled 82 articles per journal from the complete list of target articles (recall that this list contained all articles published in our focal journals, two years pre and post data availability policy change). After removing all non-empirical articles from this sample (based on empiricalness assessments by Amazon Mechanical Turkers and research assistants), our final sample had 1,222 articles. Articles in this list were randomly shuffled so that research assistants could simply work down the list, as far as possible, and ultimately code data availability for a roughly equal number of articles per journal.

Research assistants followed a relatively standardized protocol when coding data availability. For each article they coded, they first referenced the journal’s data posting policy. For those journals with straightforward policies that specify where authors should upload data, research assistants first checked for data in the mandated location. If they failed to find data in that designated location, or if they were coding data availability for journals with policies that do not designate where data should be, they conducted a broader search. This broader search entailed checking the article itself for any mentions of data, including checking data availability statements (which, while often required by journal policy, are nevertheless regularly still missing), author’s notes, footnotes, appendices, and supporting information files. Often, this broader search included scouring methods sections to check if articles used third party data sources (i.e. census data or another researcher’s dataset).

If research assistants successfully located a potential data file for an article, they then downloaded the file to read any accompanying documentation and verify existence of data files. For data files that could not be opened by research assistants (i.e. file types that could only be opened by specific proprietary computer programs that research assistants did not have), research assistants gave authors the benefit of the doubt and coded data availability as a “yes”. Likewise, if research assistants encountered an article where authors made available *some* of the data underlying their analyses but not *all* of the data, they coded data availability as a “yes”. Finally, if an article linked to an external webpage where data had been uploaded *by the author(s)*, such as an author’s personal webpage, research assistants coded data availability as a “yes” (providing the link was valid and data files were, indeed, uploaded there).

Relatedly, if articles reported using third party data but the author(s) did not upload the data themselves, either as supporting information or in another designated location such as their personal websites, research assistants coded data availability as a “no”. This was decided for the sake of streamlining our protocol. If we had decided to count data uploaded *anywhere* as a data being available, it would be nearly impossible to confidently report data availability as a “no” for any given article, since data might feasibly exist *somewhere* on the Internet.

While research assistants were coding data availability, they occasionally discovered that some articles were not actually empirical, but had been mistakenly coded as such. These articles were removed from the sample of 1,222 articles.

In total, research assistants coded 1,072 articles. Segmenting by journal and time period (pre or post policy change), Table A7 reports the number of articles coded, the number of articles with data available, and data availability percentage rates.

**Table A17: Data availability rates**

| **Journal Name** | Total articles coded | Total **pre** policy change articles coded | Total **post** policy change articles coded | **Pre** policy change articles with data | **Post** policy change articles with data | **Pre** policy change data availability rate | **Post** policy change data availability rate |
| --- | --- | --- | --- | --- | --- | --- | --- |
| **American Economic Review** | 57 | 25 | 32 | 2 | 13 | 8.00 | 40.63 |
| **American Journal of Political Science** | 74 | 61 | 13 | 31 | 10 | 50.82 | 76.92 |
| **American Political Science Review** | 57 | 34 | 23 | 8 | 2 | 23.53 | 8.70 |
| **Bulletin of the American Meteorological Society** | 61 | 36 | 25 | 1 | 4 | 2.78 | 16.00 |
| **Development** | 75 | 59 | 16 | 6 | 7 | 10.17 | 43.75 |
| **Ecological Applications** | 82 | 39 | 43 | 6 | 11 | 15.38 | 25.58 |
| **Ecological Monographs** | 80 | 42 | 38 | 9 | 18 | 21.43 | 47.37 |
| **Econometrica** | 29 ^a^ | 16 | 13 | 0 | 0 | 0.00 | 0.00 |
| **Gastroenterology** | 78 | 42 | 36 | 1 | 3 | 2.38 | 8.33 |
| **Journal of Labor Economics** | 75 | 35 | 40 | 1 | 2 | 2.86 | 5.00 |
| **Journal of Political Economy** | 55 | 26 | 29 | 0 | 3 | 0.00 | 10.34 |
| **Methods in Ecology and Evolution** | 77 | 40 | 37 | 10 | 27 | 25.00 | 72.97 |
| **PLoS Biology** | 71 | 35 | 36 | 13 | 22 | 37.14 | 61.11 |
| **Political Analysis** | 77 | 39 | 38 | 19 | 28 | 48.72 | 73.68 |
| **Proceedings of the National Academy of Sciences of the USA** | 77 | 19 | 58 | 1 | 10 | 5.26 | 17.24 |
| **Review of Economics and Statistics** | 81 | 40 | 41 | 25 | 34 | 62.50 | 82.93 |
| **Sociological Methods and Research** | 71 | 34 | 37 | 1 | 2 | 2.94 | 5.41 |

*Note*. Data availability rates are percentages.

^a^ Most Econometrica articles are theory papers, but many have mathematical formulas and even tables that may make them appear empirical at first glance, despite lacking any empirical analyses. Thus, the already small sample size of Econometrica articles was decreased even more substantially when research assistants double-checked empiricalness while coding data availability.

# Appendix B

## Christensen, Dafoe, Miguel Analysis

The CDM focuses on two prominent journals that switched their policies to require data posting: the *American Economic Review* and the *American Journal of Political Science*. We find that both journal policies were well-enforced: the AJPS policy for all articles, and the AER policy for articles from regular issues (i.e. not the annual *Papers & Proceedings* conference issue.) We focus our analysis on articles that use data (as opposed to purely theoretical articles) and those from regular peer-reviewed issues—excluding *Papers & Proceedings* issues of the AER as well as a centennial celebration issue of the APSR.

## Methods

### Data

We use data from the year of the policy change plus four years’ worth of journals before and after (2006-2014 for political science, 2001-2009 for economics). We broke data collection down into several components, searching through the text of the articles themselves and following up on any links found there, and separately searching Harvard’s Dataverse and the authors’ websites found with a Google. Each of these steps were double-coded by two independent research assistants and differences flagged and resolved. Details on each of these steps are available below.

Lastly, we searched the AER’s website itself for data and code associated with specific articles. (The journal website itself, hosted by the American Economic Association, is the location for all data and code shared by authors of published papers. See an example at <https://www.aeaweb.org/articles?id=10.1257/aer.20151260>.) We examined the name and file extension of every file associated with articles to determine if it was data or code. We did not directly examine the contents of most files, except in cases of file extensions with which we were previously unfamiliar.

Article topics were gathered using the Journal of Economic Literature (JEL) system for economics article and a simple breakdown of political science into six topics. As political science does not have an article topic coding system akin to the Journal of Economic Literature codes from economics, we coded each political science article for its topic (political theory, comparative politics, international relations, American government and politics, or political methodology) using the University of Washington’s [descriptions](https://www.polisci.washington.edu/what-political-science) by having two research assistants read the title, abstract, and if necessary, introduction. See https://www.polisci.washington.edu/what-political-science for more information. Each article was also given a data type: simulations, experimental, observational, or no data. As with the other steps, articles were double-coded, and differences resolved. A detailed Protocol is available at <https://github.com/BITSS/Citations/blob/master/data_entry/article_coding_protocol.md>.

We constructed indicators of stated, as well as actual, data and code availability, from this data. We used in-text mentions of data availability to construct a measure of *stated* availability, and successfully locating the data and code as our measure of *actual* availability. We tracked data and code availability separately, but find that most articles that share one share both. We find 528 articles that share both, 184 that share only data, and 59 that share only code. We also constructed measures of stated partial availability (one of multiple datasets, or part of the code, that could not be used to completely replicate the analysis of a paper) using the text of the article. We did not exhaustively list data used in the article and cross reference to the data available, we merely read the text surrounding the mention of data availability in the article and attempt to interpret that. “Our data and code is available..." indicates full availability, while “the data on unemployment is available..." indicates partial availability.

We gathered citations data from Thomson Reuters Web of Science (Web of Knowledge) and Elsevier’s Scopus database. Per our pre-analysis plan we had initially hoped to use Google Scholar citation counts as well, but Google goes to great lengths to block web scraping and does not, as far as we can tell, provide an API. We use Elsevier’s counts throughout most of our paper, since we have a more complete sample. However, we test our models with the Web of Knowledge counts and find very similar results.

### Details of Data Collection in CDM Analysis

We attempted to turn the data collection process into a series of small, concrete, modular steps. First, we scraped the text of AJPS and APSR articles from 2006 to 2014 from the respective journal websites using the Octoparse web application. We collected AER and QJE data from 2001 to 2009. We converted PDFs of the AER articles to text, while QJE articles were available in html format. The text for all four journals was parsed using Python to find all potential references to data availability. We searched for all URLs (using both HTML tags as well as searching for “http”, “www”, and common top-level domains such as .com, .edu, and .gov). We also searched for all occurrences of the strings “data”, “code”, “availab”, “found”, “homepage”, “website”, “webpage”, “replicat”, and “archive”. For each instance of these search terms, research assistants looked at the surrounding article text (100 characters on either side) and classified each instance in three ways:

- whether reference is partial (only a subset of the data or code) or full
- whether reference is to data, code, or both
- whether reference is a link (a URL), a name (an organization like the Census Bureau or the World Bank), or a paper.

A more detailed protocol for this step, as well as the other steps in our data collection, are available on Github. See <https://github.com/BITSS/Citations/tree/master/data_entry>. The references were classified by two independent research assistants, and any disagreements were flagged and resolved.

Any full URL links to data, code, or both, were then checked. Research assistants coded each link by hand as to whether the page could actually be loaded or was dead, and if the link was still live, whether the page actually contained the information referenced in the article text. Again, each of these links was coded independently by two research assistants and differences were resolved.

Separate and distinct from the automated search of article text, we also ran a (non-personalized) Google search of each individual author name. Research assistants evaluated the top 15 hits, trying to find and follow any links to paper authors’ personal academic websites, and tried to find data and/or code there. All website URLs were recorded. Results were double coded and differences resolved.

We also searched Harvard’s Dataverse for the combination of the title of each article AND author name (one author at a time). Resulting Dataverse entries were checked to see if Dataverse authors were the same as the article authors, and paper title matched (or was close to, ignoring the common “Replication data for” prefix on Dataverse. Two research assistants investigated each Dataverse for data and/or code availability, and differences resolved.

Lastly, we searched the AER’s website itself for data and code associated with specific articles. (The journal website itself, hosted by the American Economic Association is the location for all data and code shared by authors of published papers. An example is available at https://www.aeaweb.org/articles?id=10.1257/aer.20151260 ) We examined the name and file extension of every file associated with articles to determine if the contents was data or code. We did not directly examine the contents of most files, except in cases of file extensions with which we were previously unfamiliar.

Article topics were gathered using the Journal of Economic Literature (JEL) system for economics article and a simple breakdown of political science into six topics. We used the ProQuest Database to gather JEL codes for all economics articles. In the case that an article had multiple letter codes, as many do, we took the first. (We have not confirmed this, but it is our understanding that JEL codes after 2004 are simply assigned alphabetically and not by relevance to the article. If true, we think this is a sub-optimal policy.) As political science does not have an article topic coding system akin to the Journal of Economic Literature codes from economics, we coded each political science article for its topic (political theory, comparative politics, international relations, American government and politics, or political methodology) using the University of Washington’s [descriptions](https://www.polisci.washington.edu/what-political-science) by having two research assistants read the title, abstract, and if necessary, introduction. (See https://www.polisci.washington.edu/what-political-science for more information.) Each article was also given a data type: simulations, experimental, observational, or no data. As with the other steps, articles were double coded, and differences resolved.

We gathered citations data from Thomson Reuters Web of Science (Web of Knowledge) and Elsevier’s Scopus database. Per our pre-analysis plan we had initially hoped to use Google Scholar citation counts as well, but Google goes to great lengths to block web scraping and does not, as far as we can tell, provide an API.

### Empirical Methodology

We use the changes in journal policies as an instrument for data availability, and look at the relationship between (instrumented) data availability and citations. For political science, we collected data from all AJPS articles from 2006 through 2014 in AJPS and from APSR as a comparison group, as there was no change in editorial policy regarding data sharing at APSR during this time, and APSR provides a reasonable control group for identifying confounding temporal trends and shocks in top political science publications. For economics, we collected data on articles in the AER from 2001 to 2009, with QJE articles from the same period as a comparison group. The QJE did not adopt a data sharing requirement until 2016. (The current policy is available at <https://academic.oup.com/qje/pages/Data_Policy>.) We focus on models run with only those articles that use data, since articles without data are not subject to the data sharing policy. For political science we sometimes also drop the 2006 centennial issue of the APSR since the shorter broad think-piece essays are distinctly different from a regular issue, but results are generally not sensitive to this exclusion. Similarly, we also run models that drop the *Papers & Proceedings* issues of the AER, as they are not peer-reviewed and were not subject to the data sharing policy. We show results from regressions using the entire universe of data collected below; results are generally weaker when articles that do not use data are included.

Our estimation uses two-stage least squares regressions, as per the equations below:

| $availability_{i}=\alpha_{1}+\beta_{1}AER_{i}+\beta_{2}AJPS_{i}+\beta_{3}APSR_{i}+\beta_{4}Post{2005}_{i}+\beta_{5}Post{2010}_{i}+\beta_{6}Post{2012}_{i}+\beta_{7}AER*Post{2005}_{i}+\beta_{8}AJPS*Post{2010}_{i}+\beta_{9}AJPS*Post{2012}_{i}+\sum_{d} \sum_{t} \gamma_{td}+\nu_{i}$ |  | (1) |
| --- | --- | --- |
| $citations_{i}=\alpha_{2}+\eta_{1}AER_{i}+\eta_{2}AJPS_{i}+\eta_{3}APSR_{i}+\eta_{4}Post{2005}_{i}+\eta_{5}Post{2010}_{i}+\eta_{6}Post{2012}_{i}+\eta_{7}\hat{availability}_{i}+\sum_{d} \sum_{t} \gamma_{td}+u_{i}$ |  | (2) |

Equation 1 refers to the first stage, which predicts availability of data based on journal and publication date, while equation 2 uses predicted availability to estimate the causal impact on number of total citations. (The actual regressions shown in the paper were run in a single step with Stata 15’s ivreg2 command.) We control for the journal ($\beta_{1},\beta_{2},\beta_{3})$, date of publication (whether it is after the policy: $\beta_{4},\beta_{5},\beta_{6}$), and our instruments are the interactions ($\beta_{7},\beta_{8},\beta_{9}$). We also flexibly account for time since publication (and the fact that total citations mechanically increase over time) with fixed effects for each discipline-year combination ($\gamma_{td}$). We have also run specifications with a cubic function of months since publication, results are essentially unchanged.

The main concern with our empirical approach is whether the change in editorial policy is in fact independent of other causes of citations. If unobserved characteristics of quality changed as a result of the change in editor, aside from the change in data sharing policy, then the exclusion restriction in our instrumental variables method is violated and estimates may be biased. One way to test this assumption, which we do below, is to look at the observable characteristics of articles and see whether these were affected by the editorial changes. If not, we can be more confident that unobservable characteristics are also uncorrelated with editorial changes.

To do this, we classified articles by research method (experimental, observational, theoretical), by subject matter (American politics, public policy, international relations, comparative politics, political methodology, and political theory for political science and by JEL code for economics), and by the top institutional ranking of the authors. (We originally proposed to use the average 2013 *US News & World Report* Political Science PhD rankings for political science, which we deviated from in two ways. First, we were unaware that new rankings would be completed before our data collection and we opted for the newest rankings, though rankings are very stable over time. Second, we opted for the top ranking of any of the authors instead of the average, since many institutions are unranked making averages complicated. 2017 political science rankings are available at <http://grad-schools.usnews.rankingsandreviews.com/best-graduate-schools/top-humanities-schools/political-science-rankings> and economics rankings are available at <https://www.usnews.com/best-graduate-schools/top-humanities-schools/economics-rankings>.) We then used our same specification as in equation 1, but with these variables as the outcome.

## Results

### Summary Stats

We collected data on 3,000 articles, two-thirds of which are economics articles. Excluding articles without data, *Papers & Proceedings* and centennial issues leave us with 1661 articles, 55 percent of which are economics. We present summary statistics of these 1661 articles in Table B1. (Summary statistics for the full sample are in Appendix Table B20.) The majority (68 percent) of economics articles are from the AER, as the QJE publishes far fewer articles per year. The AJPS also publishes more articles than the APSR. Economics and political science publish similar proportions of articles by data type: 13 to 15 percent experimental, over 80 percent observational, and fewer than three percent simulations. (25 percent of the complete sample have no data.) Economics as a discipline is very top heavy—the top economics journals publish a much higher fraction of articles from the top ranked institutions than political science: 37 percent in economics compared to 14 percent in political science, where top ranked is defined as the top six universities in either discipline. (We pick six to count the same number of universities for each discipline: economics has six universities tied for number one, while political science has two, but also three universities tied for fourth.) Data sharing is higher in political science than in economics: 36 percent compared to 25 percent for data and code, or 44 percent compared to 38 percent for just data. Lastly, citations are higher for the average economics articles than in political science, 167 compared to 52.

**Figure B1** The distribution of citations, including regular data articles with fewer than 500 citations.

Figure B1 shows that the distribution of citations across articles is highly skewed. (For interpretability, we have cut the figure off at 500 citations; there is one political science article beyond this, while there are 158 economics articles.) The median economics article has 99 total citations, the median political science article has 33 total citations, and the median article in the combined sample has 61 total citations. Some of this difference is explained by the earlier time period of the economics articles. Overall, citation rates are similar on a per-year-since-published basis: the economics articles have 8 citations per year on average while political science articles have 5.

**Table B1: Summary Statistics**


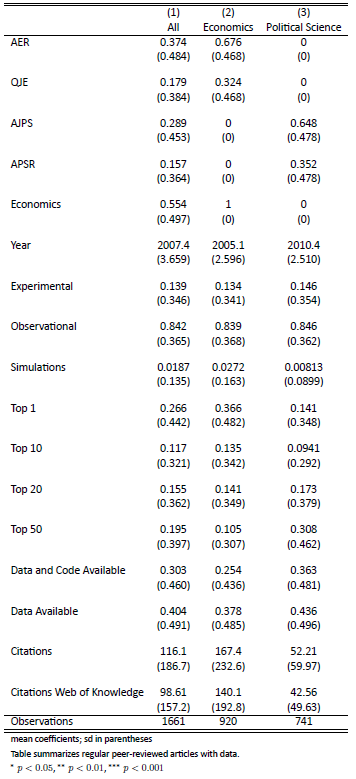


We also show the total accumulated citations of articles over time (averaged across all articles in a year in each journal) in Figure B2. While the AER, APSR, and AJPS appear to have similar rates of citation accrual, articles in the QJE clearly acquire more citations per year (13) on average.


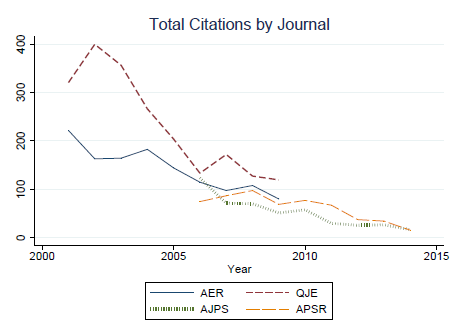


**Figure B2** Citations over Time. Figure shows total cumulative citations as of November 2017 based on year of article publication.

A similarity across the disciplines appears to be the strength of enforcement of the policy, which we summarize in Figure B3. The figure shows the fraction of articles with both data and code made publicly available in the top row, and (at least) data made publicly available in the bottom row—the patterns are broadly similar, though by definition sharing is at least as high as data and code, and sometimes as much as 20 percent higher. To get a clearer sense of the degree of enforcement we limit the sample of articles to only those that use data (for example, dropping pure math or pure theory articles without empirical tests) and in economics, we also drop the articles from the annual *Papers & Proceedings* articles. These shorter articles from the annual May issue are presented at the annual American Economic Association conference in January and appear not to be subject to the data sharing policy, as none of them have data on the AER website. In political science we see an obvious effect from the policy. At the AJPS, before any policy was instituted, data sharing was about 20 percent. After 2010, it jumped to 40 percent, and after 2012 it was over 80 percent. In economics, data sharing increases from 20 percent in years before the policy to 60-80 percent after the policy. While imperfect compliance is a possibility, another partial explanation is potentially the use of proprietary data. The annual editors report of the AER contains a summary of how many articles request the exemption, though it does not identify individual articles. This fraction has risen dramatically from 6 percent in 2005 to over 40 percent at present.


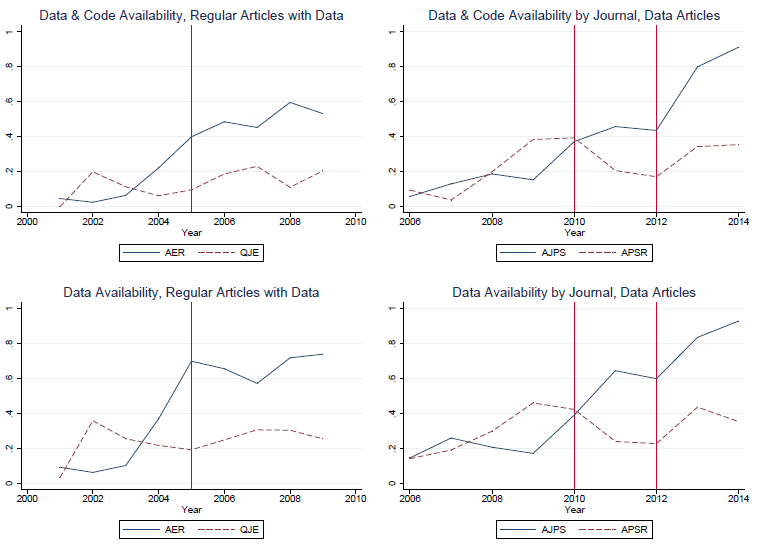


**Figure B3:** Data and Code Availability over Time

### Naive OLS Results

We first investigate whether we find similar broad associations in economics and political science as others have found in other disciplines. Tables B2 and B3 summarize these results. We regress total number of citations on whether data and code is available. Regressions on whether data alone (i.e. at least data regardless of code) is shared are in Tables B13 and B14 and are similar but show even stronger associations. Note that in all tables in this appendix section B, the sample descriptions listed as “Data-Only” imply using only articles that use data. “Data-NoPP” implies a further limitation of removing the *Papers & Proceedings* conference and similar anniversary-style issues. “Data-Econ” or “Data-PolSci” imply limiting to only data articles from the respective discipline, and “Data-NoPP-Econ” or “Data-NoPP-PolSci” is only data articles in regular issues from the respective discipline. The first specification includes all articles that use data, and the second, which is our preferred specification, limits the sample further by excluding articles and the APSR Centennial issue. (See Tables B20 through B23 for specifications using articles that do not use data, and for the political science sample only.) We control for journal of publication and time since publication in the form of year-discipline fixed effects. We have also tested models with a cubic function for months since publication—another way to flexibly account for the mechanical accumulation of citations over time—and results are nearly identical.

**Table B2: Naïve Ordinary Least Squares Regression**


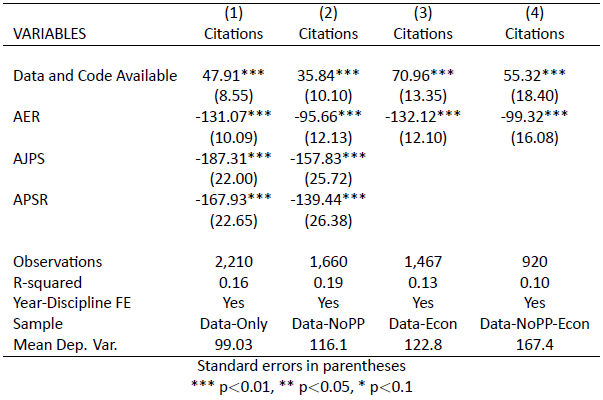


**Table B3: Naïve Ordinary Least Squares Regression, Log Citations**


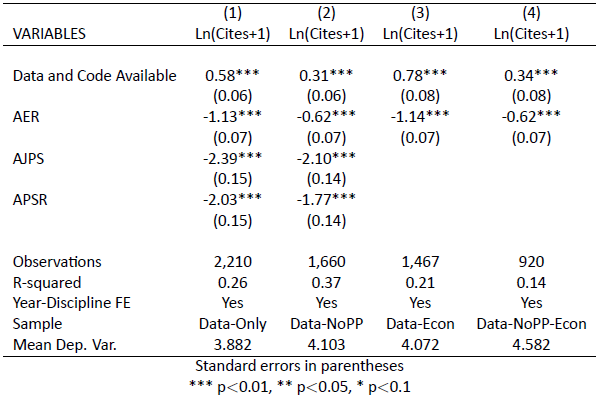


The regressions show a consistently positive association between citations and the sharing of data and code. On average, articles with data and code available have 36 to 48 extra citations, compared to an overall mean citations rate of 99 to 116 citations. This large percentage association is also shown in Table B3, where we run the same specifications, only using the natural log of citations plus one as the outcome. Here the coefficient of .34 represents 40 percent higher citations for articles that share their data and code.

The third and fourth columns show the same regressions with the sample limited to economics articles. Associations between data sharing and citations are slightly stronger. Of course the results presented here do not account for endogenous selection into data sharing, which we attempt to do below.

### Two Stage Least Squares

Next, we implement the two-stage least squares regressions, instrumenting for data availability with whether an article is published after the policy is implemented in that journal. We show results in Tables B4 and B5. The first and second specifications use both disciplines. The first limits the sample to only articles that use data, while the second removes P&P and APSR centennial articles. We use simple binary indicators for whether the article was published in the data-requiring journal after the policy was implemented for instruments (after 2005 in the AER, for example). Below we show regressions on the full sample of articles including those without data, and we also test a different instrument to attempt to isolate the effect to articles that use data—in that case we instrument with the journal policies as well as the interaction of the article using data and being after the policy in the policy journal. We also expand the instruments even more to include the interaction of a data article and a non-P&P article for the AER.The third and fourth specifications use the same instrument (publication in the policy journal after the policy) but limit the sample to only economics articles. Across both disciplines, sharing data and code appears to cause an increase of 91 to 126 citations, off a mean of 99 to 116 citations. The economics-only specifications produce an implausibly large estimate when P&P articles are included, but a more reasonable doubling of citations when they are excluded. The large coefficient in the third specification is likely due to the weak first stage in that specification, which we discuss below. Political science-only regressions show a strong first stage, with F-statistics of 22, but smaller and imprecisely estimated effects of data sharing on citations ranging from four to eight citations. See Table B21 for political science 2SLS results and Table B14 for complete first stage results.

**Table B4: 2SLS Regression**
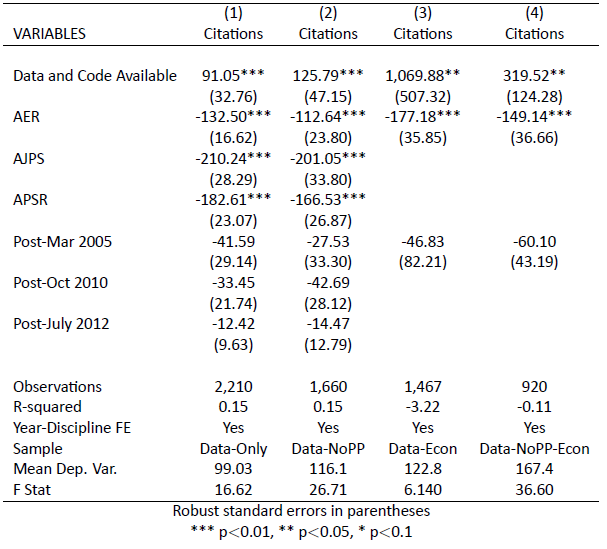


**Table B5: 2SLS Regression, Log**
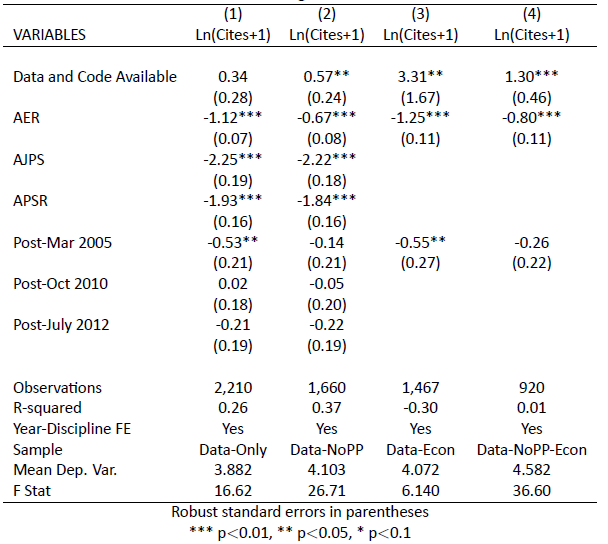


We produce the analogous natural log estimates in Table B5. Estimates are still large but slightly smaller than the linear model. The specification without P&P and Centennial issues indicates $\alpha<.05$ with a coefficient of .57 corresponding to a 77 percent increase in citations. We again show these same regressions for only data sharing (as opposed to data and code) in Tables B6 and B7; results are similar.

**Table B6: Data-Only Sharing, 2SLS**
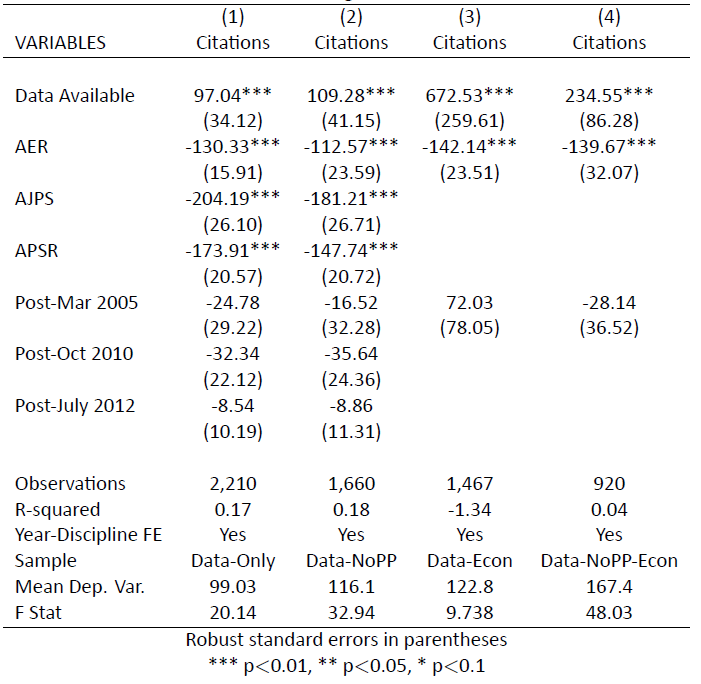


**Table B7: Data-Only Sharing, 2SLS Log**
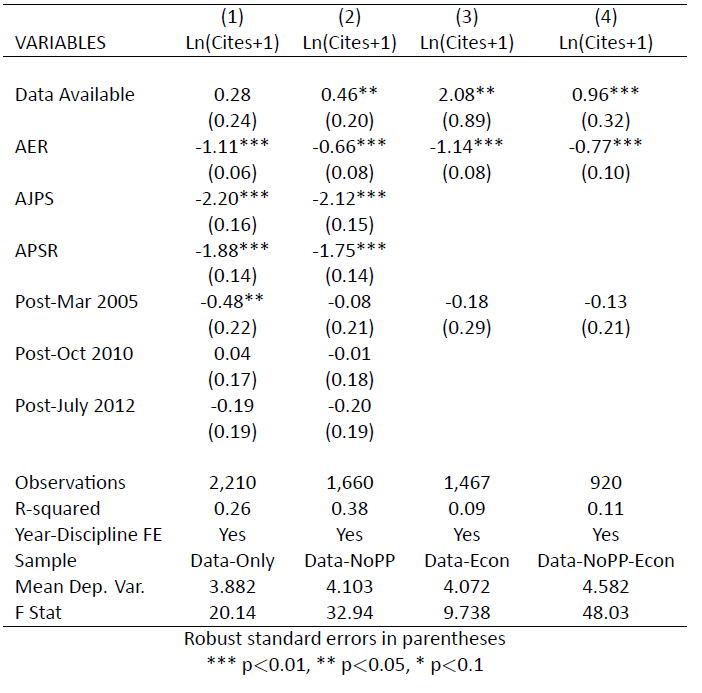


It is necessary to test the strength of the first stage when using two stage least squares methods. In our case, this is a test of how strongly the journal data sharing requirements were enforced compared to how low data sharing was prior to the policy, and how low data sharing was, and remained, low in the comparison journals. F statistics are included beneath each specification. Using both disciplines, they range from 17 to 27, so we conclude that we are free from problems of weak instruments in the combined sample. The complete first stage regression is shown in Table B8, and the results for data sharing only (as opposed to data and code sharing) are shown later in Table B15.

**Table B8: First Stage Regression Results**

**
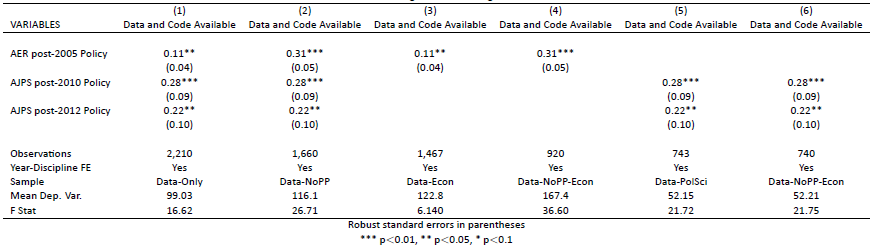
**

### Exclusion Restriction

One of the main assumptions of the instrumental variables method is the exclusion restriction—the assumption that the instrument only affects the outcome through the selected endogenous channel, and not through any other unobservable way. In our case, this means that the journal data sharing requirements cannot have changed the type of articles that were being submitted to the journals except for the change in the likelihood of data and code being made available. If certain types of authors changed their journal submission habits, or submitted fewer empirical articles to avoid the policy, or because they were pleased with it, this could bias our estimates.

One especially plausible way this could happen is due to the change in editor. This is a potential concern in both cases, as Rick Wilson instituted his policy soon after becoming editor, and Ben Bernanke edited the AER from 2001-2004, instituting the policy toward the end of his tenure. The editorial board at APSR also changed in 2012, indicating that other characteristics of articles may have changed there as well. See list of AER editors at <https://www.aeaweb.org/journals/aer/about-aer/editors>, and the announcement of the APSR editorial change at <https://politicalscience.unt.edu/apsr>. We show some of the data on articles in Figure B4, separately for economics and for political science. The top row shows article topics, the second article data types, and the third row the ranking of author institutions. (One unfortunate fact clear in this figure is that a rather implausibly high fraction of political science articles are classified as methodology. This pattern of article classification is at least internally consistent across journals and thus unlikely to drive our results.) These figures include all articles, including those that do not use data, since whether or not articles use data or rely on theory is an important characteristic to test.


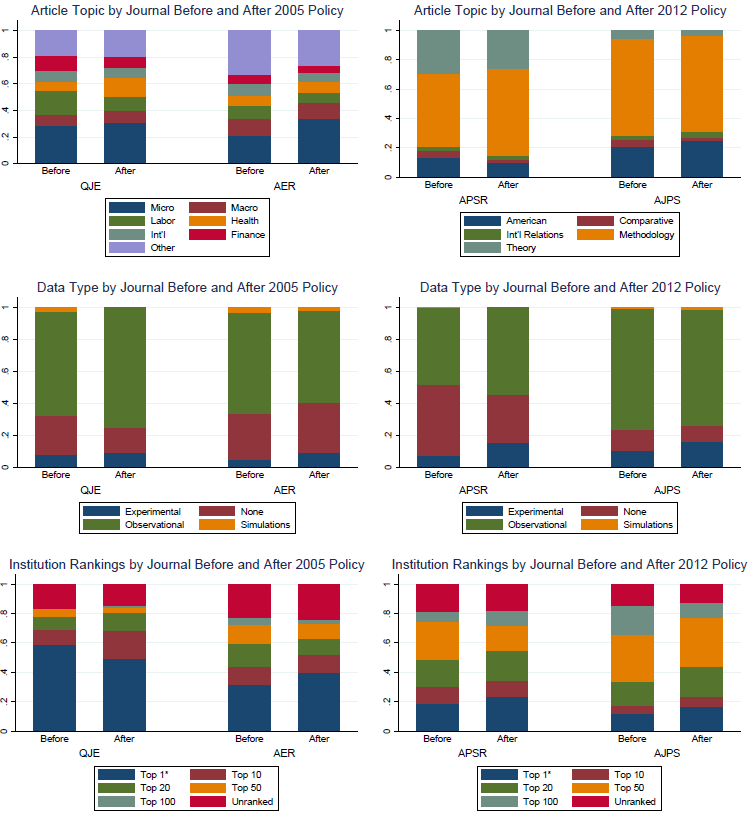


**Figure B4:** Article Topics, Data Types, and Author Institution Rankings

The assumption is not perfectly testable, but we can test it to a certain extent using the same specification as in our first stage and checking if any observable factors did indeed change. We do this with the data on article topic, data type, and institution ranking. Results are shown in Table B9. We examine the combined data from both disciplines using our preferred specification of only data and non-P&P articles, and see that there seems to be some shift towards experimental papers away from observational work, as well as more publication of top-ranked authors, correlated with the AER policy change. Note again that we classify the top six universities as top-ranked to deal with ties. Though we believe there was likely a secular trend towards more experimental work at this time, this would not explain why more of these would be published in the AER relative to the QJE.

**Table B9: Test of Exclusion Restriction, Combined Disciplines**


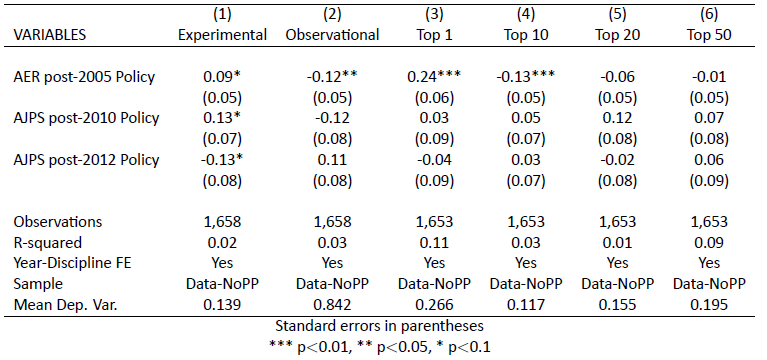


Since this is indeed a concern for the validity of our instruments, we present the results from each discipline separately in Table B10. The top segment shows regressions on the division of economic topics (JEL codes), with an apparent increase in the proportion of labor economics papers: an eight percent point increase off a base of ten percent. We show the six largest JEL codes representing over 70% of articles. Not shown is a nearly as large but opposite (and insignificant) decline in all other combined categories. We also see an increase in experimental papers (10 percent) and fewer observational papers (12 percent). There is also an increase in the fraction of papers coming from the universities tied for the top ranking. However, none of these patterns are repeated with political science. This makes interpretation of our results more difficult. If experimental articles and those with authors from top-ranked institutions are likely to be cited more regardless of data sharing, as seems plausible, our estimates would be biased upwards. We also test a version of our instrumental variables model in which we control for the characteristics of articles that change: whether the article is experimental or observational, has a topic of labor economics, and is written by top-ranked or top-10 institution authors. This specification implicitly assumes that while these characteristics of articles may change with the policy, no others do. It indicates an increase of 115 citations, similar to our previous estimates. See Appendix Table B22 and B23 for details.

**Table B10: Tests of the Exclusion Restriction by Discipline**
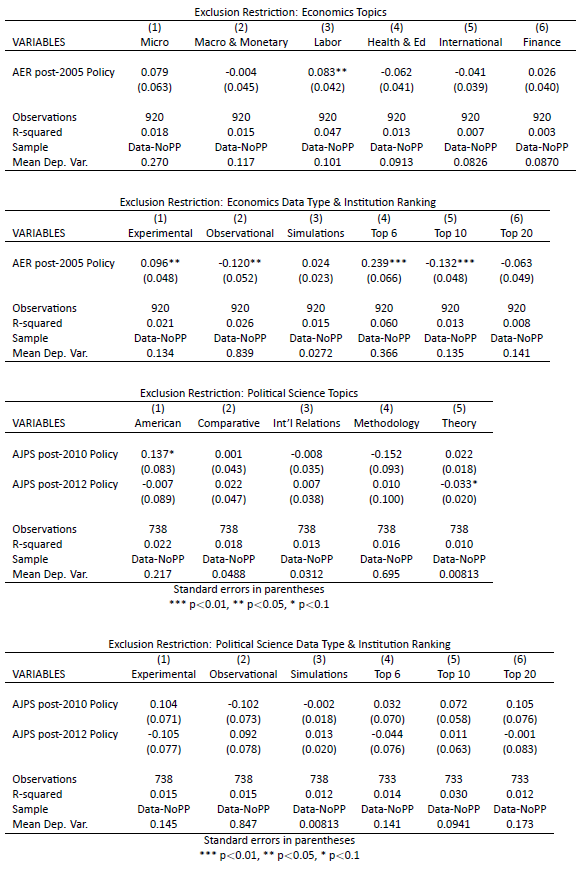


### Different Measures of Availability and Citations

Searching the text of every article gives us the ability to evaluate stated versus actual availability. We classified in-text mentions of data being available and whether that data was available on a specific website, Dataverse, from another paper, or from an organization such as the Census Bureau or World Bank. We also recorded data on whether data or code was *partially* available. Journal policies make clear that entire datasets and complete code should be shared, but sometimes authors refer to a subset of their data being public, or describe the ultimate source of the data. So, we recorded “The outcome variable is available from the World Bank" as partial data availability. However, the first stage of these regressions are weak, F statistics for the instruments are never higher than four, implying that the policy had little effect on inclusion of statements like this. 2SLS results vary dramatically depending on specification. We do not observe a strong relationship between this measure and citations. In total, we found data and code for 503 articles. There were 310 articles that stated their data and code was available. Of those, we found 199 or 64 percent. Instrumenting for stated (instead of actual) availability we find a very strong first stage, with F statistics as high as 100. This appears to be driven mostly by the 2010 AJPS policy, which explicitly required stating where the data could be found. However, the 2SLS regressions show small, mostly negative, and insignificant effects of stated availability—the preferred specification indicates a decrease of seven citations but the estimate has a standard error of 12. We show these results in Table B11. Political science results appear below in the section of results for all articles.

**Table B11: Stated Availability, 2SLS Regression**
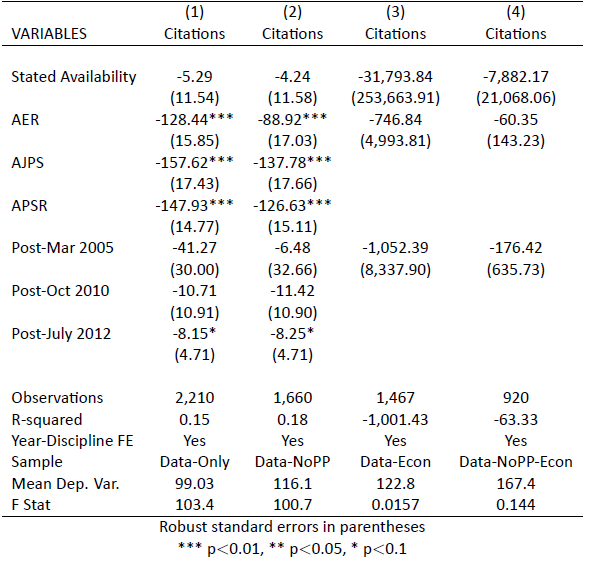


We also investigate the flexibility in our choice of citation count. The correlation between the two sets of citations is quite high, over .99. Though the data was collected within a month of each other, Scopus citations are greater in 95% of the articles for which we were able to find a citation count in both sources. Figure B5 shows the very strong relationship between the citation counts. Results using the alternate Web of Knowledge citation counts provide very similar results to those shown in the paper—our main specification indicates an increase of 89 citations from sharing data. We show our main 2SLS results with this alternate outcome measure in Table B12.


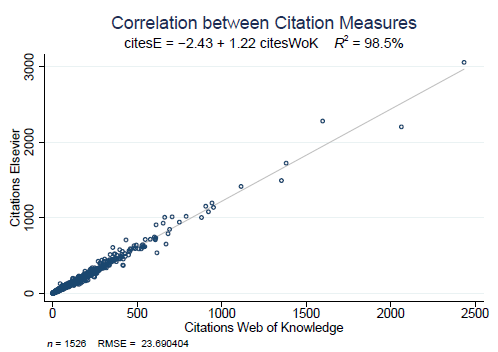


**Figure B5:** Figure shows correlation between two main data sources for total citations as of November 2017.

**Table B12: Alternate Citations Measure, 2SLS**


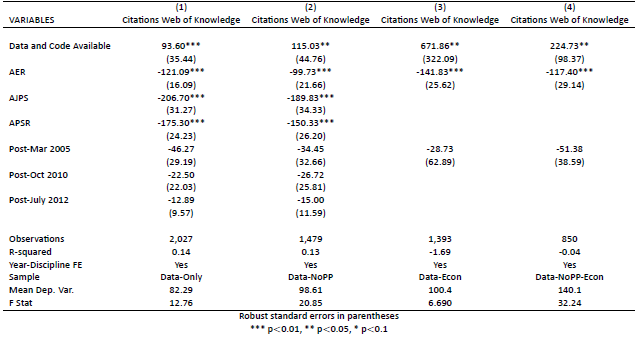


### Results for Data Availability Only

In this section, we provide tables analogous to those above, only we use data availability instead of data and code availability. In order, these are the OLS results (linear and log), the 2SLS results (linear and log), first stage results, tests of the exclusion restriction for combined disciplines, and the 2SLS results with the alternate source of citations data. We also show residual versus fitted plots for the main OLS and 2SLS regressions for data availability in Figure B6.

**Table B13: Data-Only Sharing, Naïve OLS Estimates**
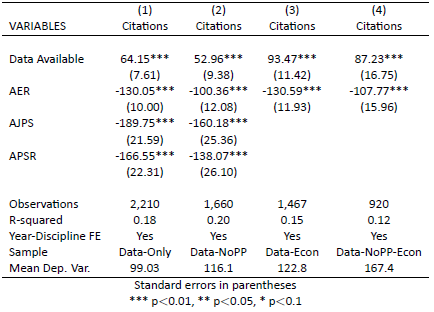


**Table B14: Data-Only Sharing, Naïve OLS Log Estimates**


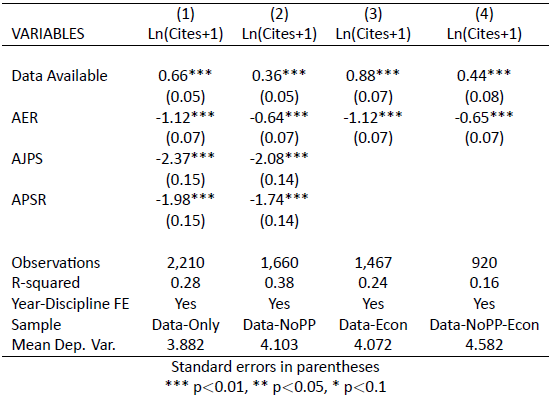


**Table B15: Data-Only Sharing, 2SLS Regression**
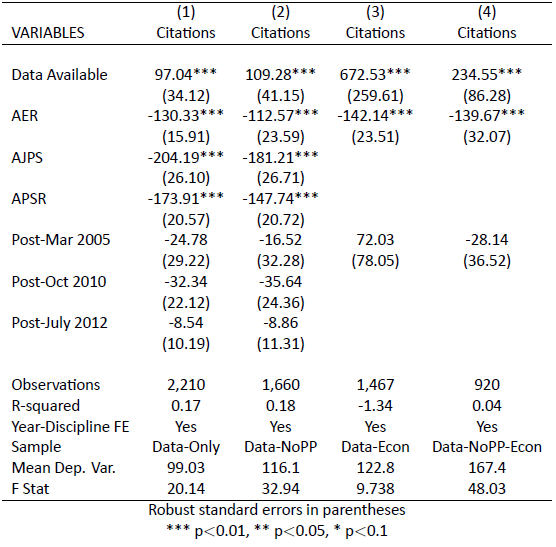


**Table B16: Data-Only Sharing, 2SLS Log Regression**
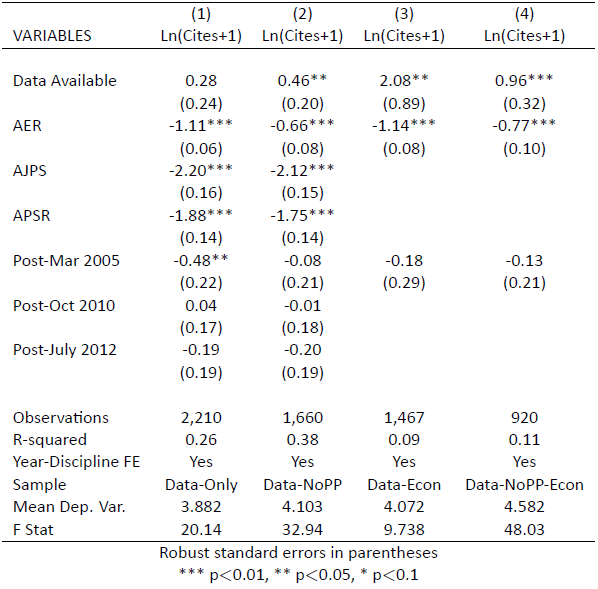


**Table B17: Data-Only Sharing First Stage Regression**


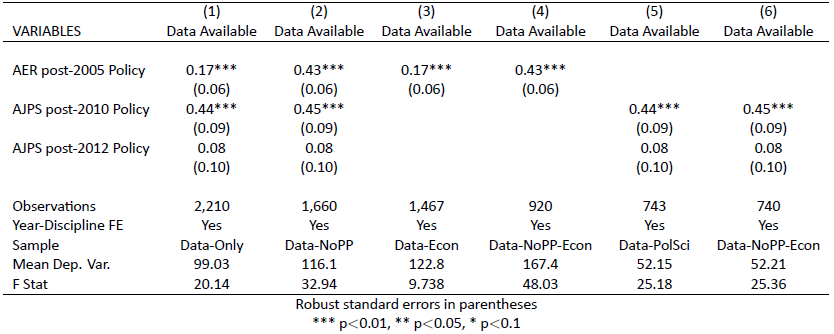


**Table B18: Data-Only Sharing, Exclusion Restriction, Combined Disciplines**
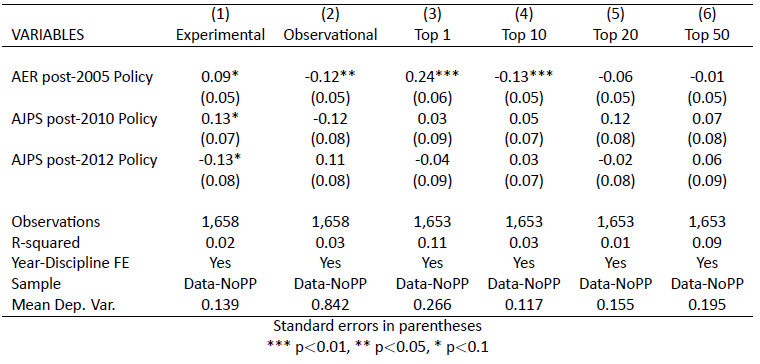


**Table B19: Data-Only Sharing, Alternate Citations Measure, 2SLS Regression**


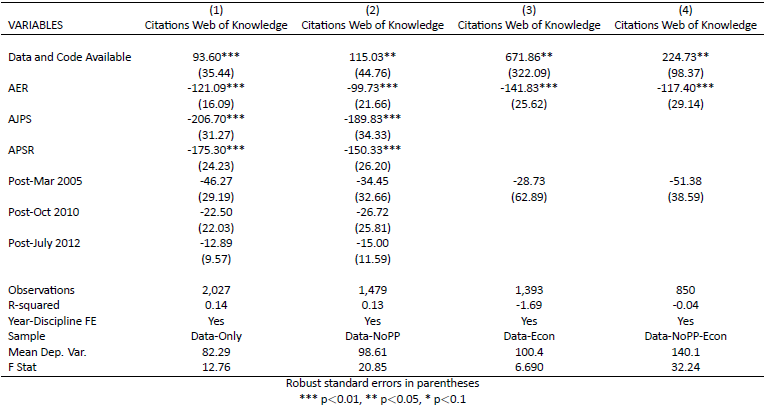


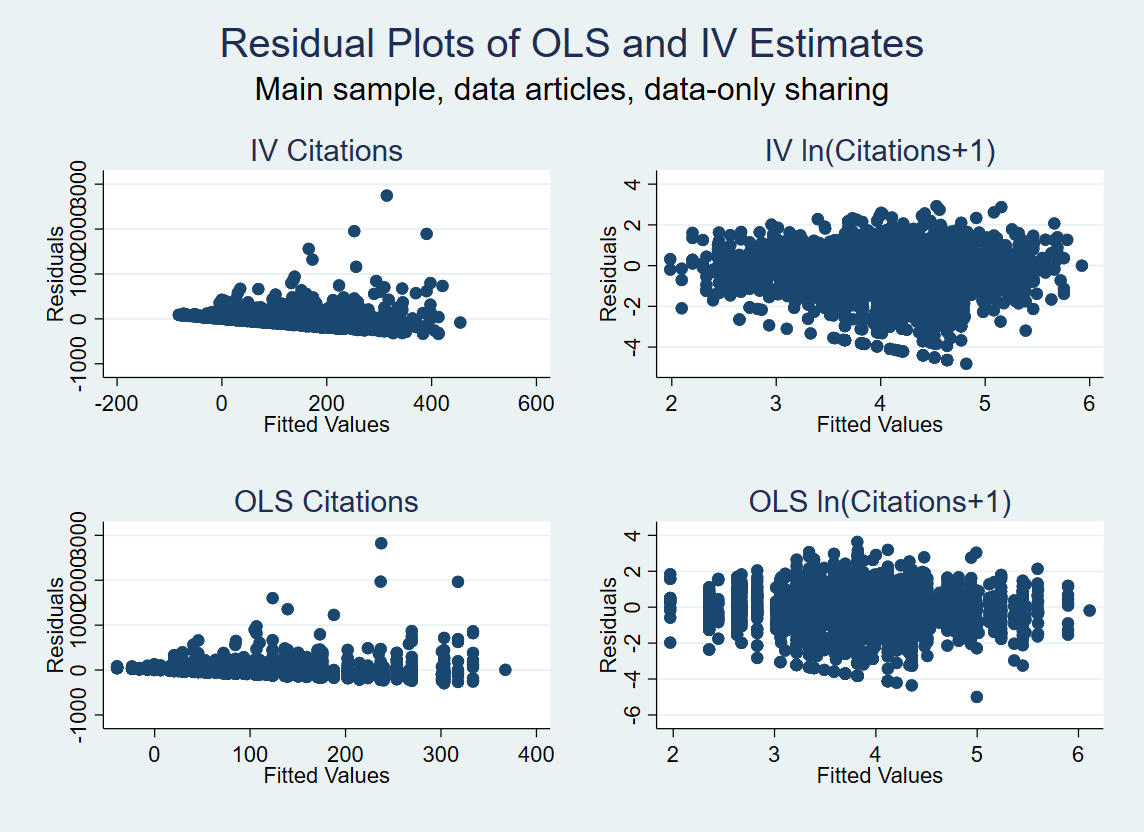


**Figure B6:** Residual versus fitted plots for the sharing of data only, for linear and log regressions, for OLS and 2SLS main sample specifications (non-conference proceedings regular empirical articles from both economics and political science).

###

### Results for All Articles and for Political Science Only

For completeness, we show analogous figures and tables but for the full sample of all articles, which includes papers without any data that would likely not be affected by any change in data sharing policy, *Papers & Proceedings* articles from the AER, and the Centennial issue of the APSR. We show summary statistics in Table B20, the OLS regression in Table B21, the 2SLS regression in Table B22, and the stated availability outcome in Table B23. We also control for whether the article has data in it, and in some specifications (columns two and three of Table B22 and B23), create additional instruments by interacting being in a treatment journal post treatment with being a data article. The fourth and fifth specifications show regressions for data articles, but with only political science.


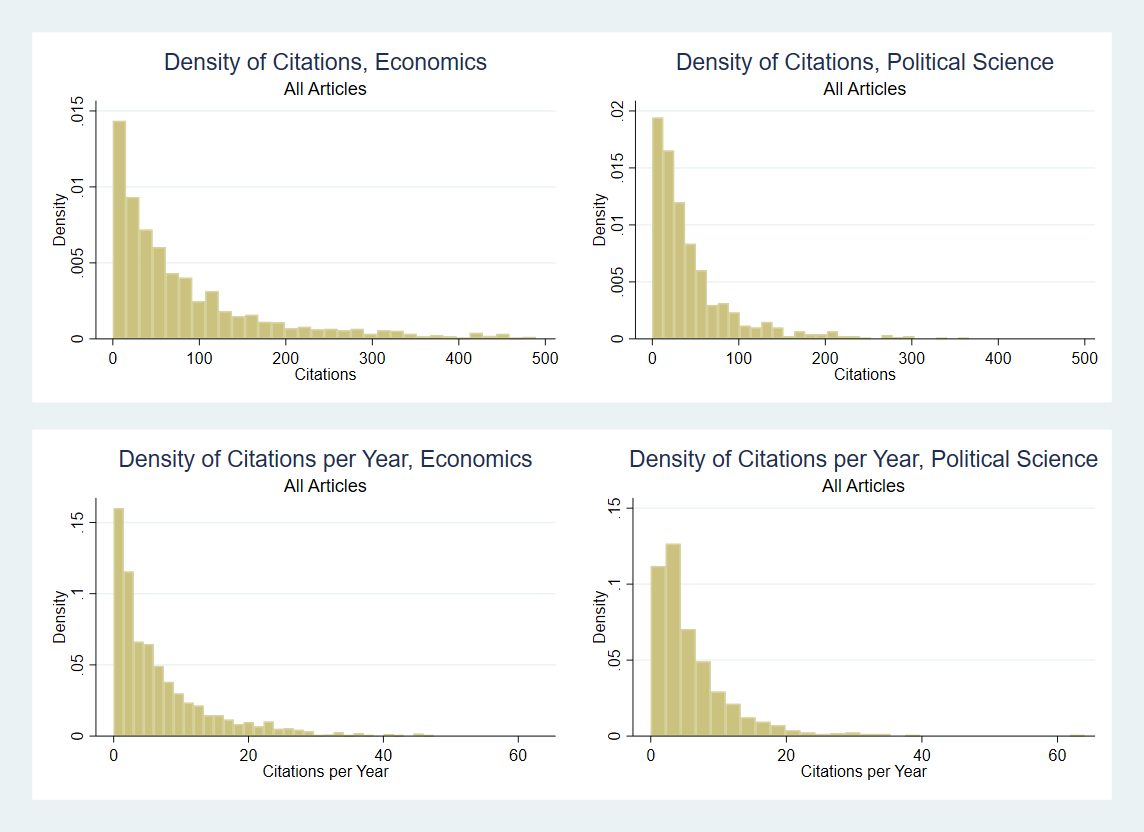


**Figure B7:** Histograms of citations for all articles with fewer than 500 citations

**Table B20:** Summary Statistics for All Articles
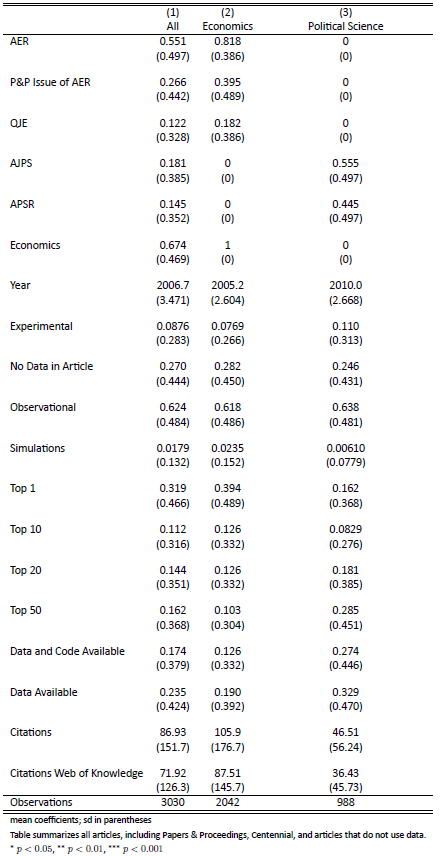


**Table B21:** Naïve OLS Regression for All Articles and Political Science Only
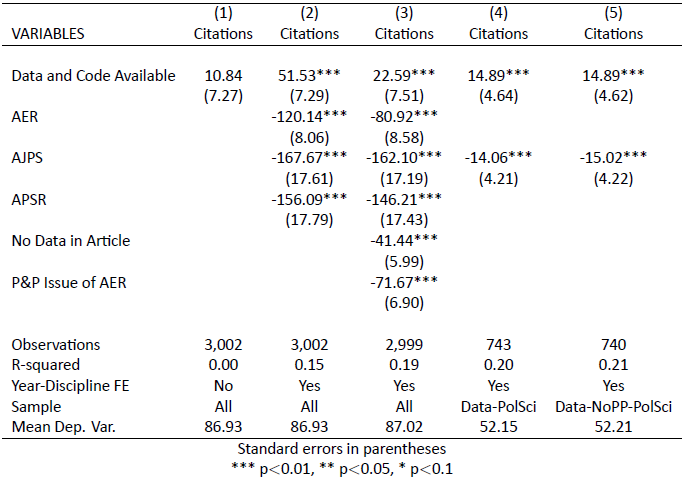


**Table B22:** 2SLS Regression for All Articles and Political Science Only
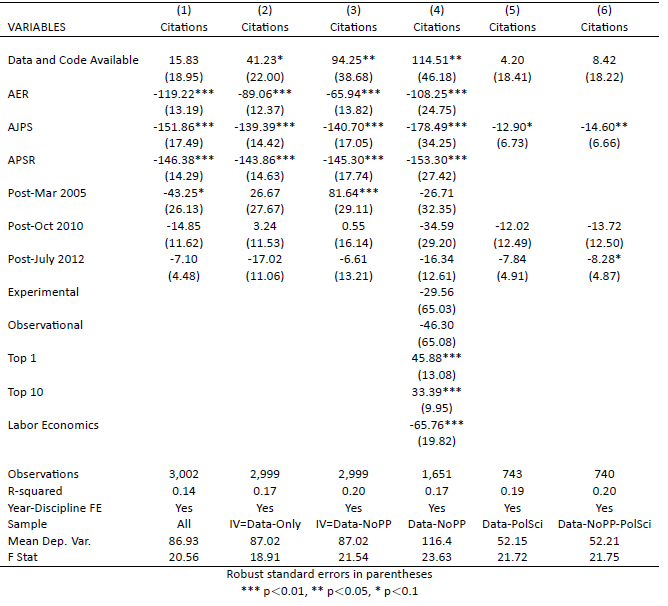


**Table B23:** Stated Availability 2SLS Regression for All Article and Political Science Only
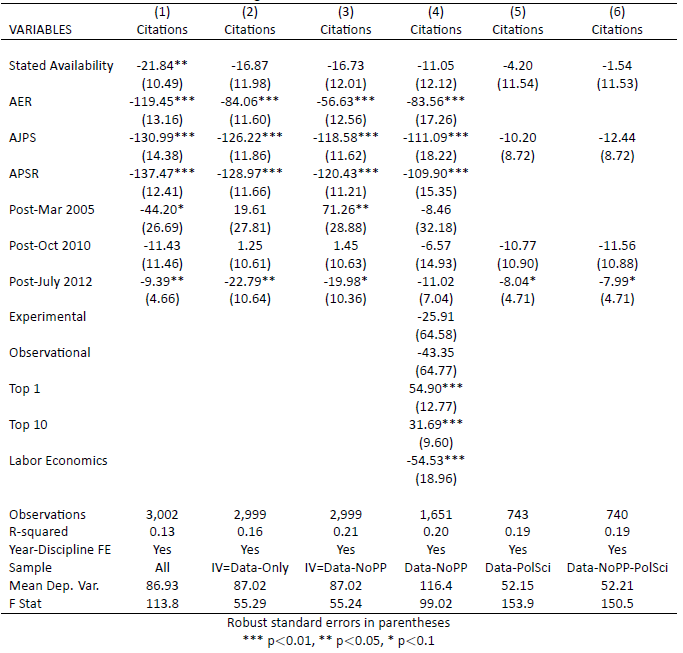


# References

1. Casey K, Glennerster R, Miguel E. Reshaping Institutions: Evidence on Aid Impacts Using a Preanalysis Plan. Q J Econ. 2012 Nov 1;127(4):1755–812.

2. Humphreys M, Sierra RS de la, Windt P van der. Fishing, Commitment, and Communication: A Proposal for Comprehensive Nonbinding Research Registration. Polit Anal. 2013 Jan 1;21(1):1–20.

3. Dafoe A. Science Deserves Better: The Imperative to Share Complete Replication Files. PS Polit Sci Polit. 2014 Jan;47(01):60–66.
