# Supplementary material for: A study of the impact of data sharing on article citations using journal policies as a natural experiment
Source: PLoS One. 2019 Dec 18;14(12):e0225883. doi: 10.1371/journal.pone.0225883 (PMC6919593; doi:10.1371/journal.pone.0225883)
Supplement: Attachment — Submitted filename: 191107 ResponsetoReviewers.docx [file pone.0225883.s003.docx]

November 3, 2019

Response to Reviewers

Thank you to our reviewers for their continued careful review of our paper. We respond point by point to the comments that were highlighted by the editor. The initial reviewer comments are in italics, while our responses follow in regular typeface.

*In addition, in your abstract, please provide explicitly more details about your methods and results, expressed in a quantitative way using point estimates and 95 % confidence interval for your main outcomes.*

Thank you for this suggestion. We now specifically mention both of our main methods (matching, instrumental variables) and describe the results quantitatively, reporting the standard error of our main estimate, as is reported in the tables and as is customary in our disciplines of economics and political science.

*Please also make a short statement of your main limitations (see below) in the introduction.*

The last sentences of the introduction now discuss our generalizability and limitations (namely, that we cannot address *why* articles are cited more, and we use observational not experimental data.)

*- In the method section, please provide all the details necessary to reproduce your selection of journals. Please try to adapt the PICOS formulation of the PRISMA statement for this. Please provide more details concerning the matching of journals explaining specifically why 17 journals were matched with 13 journals (this should be also addressed in the discussion section).*

We now clearly address this in the methods section with the following language:

“To account for the possibility of events that influenced both the change in journal policy and citation rates that could bias estimates, we collected comparable data for two natural comparison sets.  We gathered data on *theoretical* articles published in the same journals; since these do not use empirical data, their citations should be largely unaffected by any change in data posting policy.  We also matched the 17 *treatment journals* (which began to require data sharing) to *control journals* (which did not), and collected comparable citation data for empirical articles published in these control journals.

Our control journals are derived using conventional one-to-one propensity score matching with replacement from top-ranked journals that most closely match the treatment journals on six SCImago criteria (8,9).  Our objective was to identify control journals that did not require data posting, but that were otherwise as similar as possible to the treatment journals in terms of observable characteristics.  Accordingly, we began with non-treatment journals -- that never required data posting – from the same SCImago “Top 250” list of journals where we searched for our treatment journals. We matched treatment to control journals using the six indicators used to create the SCImago list itself.  These criteria vary by journal and are posted by Scimago on its website.  The six variables are: a) the journal’s h-index; b) the total number of citable documents published in the journal’s last three years; c) citations per document over the last two years; d) references per document; e) the country where the journal was published; and f) the category of the journal.  Since there are only two countries where our treatment journals were published, we created a binary variable for journals published in the UK, leaving US journals as the default.  And since we only have a limited number of treatment journals, we consolidated journal category into eight areas: a) Biology; b) Ecology; c) Economics; d) Medicine; e) Molecular Biology; f) Multidisciplinary; g) Sociology and Political Science; and h) Miscellaneous.

After creating the data set, we created a binary variable, with unity for our treatment journals and zero for all remaining journals (potential control journals).  We then estimated a cross-sectional probit equation; the results are tabulated in our online supplement.  After estimating the probit model, we then matched each of the treatment journals to a single control journal, using the closest possible journal, by predicted probit score within journal category.  Sometimes this resulted in more than one treatment journal being matched to the same control journal.  Both the control journals and the probit regression estimates themselves are freely available online.  We are left with a set of 13 unique control journals along with 17 treatment journals; thus the MR analysis included data from a total of 30 distinct scientific journals.  Appendix A provides the list of journals and more details on the data construction procedure.”

*Please make a specific paragraph "changes to the initial protocol".*

This information was already explicitly included in the appendix, but we now include it in the main methods section as well, as follows:

“In 2015, the CDM team pre-registered their analysis (<https://osf.io/hv97m/>). We deviate from it here in the following ways: First, we did not think to exclude articles that do not use data. (Results on the full sample, described in Appendix Tables B21 to B23, are generally weaker.) We also planned to control for time using cubic functions of months since publication. The results presented in the paper instead use year-discipline fixed effects to more flexibly control for time, as can be seen in the equations below. (Models with the cubic function yield nearly identical results.) In September 2017 CDM, expanded their analysis from the pre-registered field of political science (from which they had seen results) to include the economics portion of the project; this was before seeing any results from the broad analysis conducted by DM.”

*Please use subheadings to differentiate the different parts of the project (e.g. broad analysis / deep analysis / and a specific part statistical analysis).*

This is a good suggestion that will help make our paper clearer. We have added subsections to both methods and results for both the broad analysis and deep analysis. As both of these analyses are clearly statistical in nature, we do not specifically label either as statistical.

*Please do not use the word "causal" in the manuscript, as you are only exploring association and not causal relationships : e.g. when you state : "equation 2 uses predicted data availability to estimate the causal...") ;*

This was the only instance of the word “causal” in our paper. We are happy to modify the language here to say that “equation 2 uses predicted data availability to attempt to estimate the impact on number of total citations under assumptions we discuss below,” which we believe is completely accurate. We have also added citations to the 1996 Angrist, Imbens, and Rubin JASA paper “Identification of Causal Effects Using Instrumental Variables” (which has been cited over 5,000 times) and the 1994 Imbens and Angrist *Econometrica* paper “Identification and Estimation of Local Average Treatment Effects” (cited over 4,400 times) which both strongly make the case that instrumental variables can be used to estimate causal effects under certain assumptions. This is in addition to the Dehijia and Wahba (2002) *REStat* paper with 4,600 citations and the Rosenbaum and Rubin (1985) paper with 23,800 citations that discuss propensity score matching and causality, both of which we already cited. We discuss and test these assumptions in our paper to the extent possible, and are forthright about the evidence that we may violate one of these assumptions. We thus feel strongly that it is appropriate to state that we *attempted* to estimate causal effects, because that is, in fact, what we attempted to do.

*- In the result section, please provide all quantitative results of the model described in the method section. Importantly, I would expect you to report point estimates and 95 % CI. Table(s), or results written in the text must appear in the paper. As it is now, there are only qualitative descriptions/informations about the results. This section must report data, be factual and should not present a discussion of the results. Please move any interpretation to the discussion section.*

Thank you for this suggestion. We have checked that every numerical result in the paper is either:

(a) directly from a table that appears in the paper itself, if a main result, with standard errors included

(b) from a discussion of a figure, and thus not an exact statistical result, and thus not possessing a standard error

(c) from a table that appears in the appendix, if not a main result and instead the result of a robustness check, with the exact appendix table number specified.

We have moved additional results tables into the main paper, but in order to be transparent and exhaustive, the appendix includes 40 tables, and certainly not all of these belong in the main paper.

*- In the discussion section, please start with a description of the principal finding and here please interpret your findings. Please add an important emphasis on risk of bias and especially confounding. Please make it very clear that this study is about associations and not causal relationships. Please also discuss the rational and the previous literature about the expected association between citations and data sharing practices. Please try to be systematic and exhaustive in your appraisal of the literature.*

Thank you for these suggestions. We now begin the discussion section with a discussion of our main results. We discuss bias in both of the methods sections, and have lengthened the discussion regarding both of the main methods, their assumptions, and potential biases.

Though this article is not intended as a review piece, an updated search did provide a few extra citations, which we discuss in more detail. If you are aware of others that we have not identified, we are open to suggestions. Regarding the interpretation of our results, and in particular whether we are estimating causal impacts, please refer to our discussion above of your earlier point.

Sincerely,

Garret Christensen, Allan Dafoe, Edward Miguel, Don A. Moore, and Andrew K. Rose
